# De-Novo Discovery of Differentially Abundant Transcription Factor Binding Sites Including Their Positional Preference

**DOI:** 10.1371/journal.pcbi.1001070

**Published:** 2011-02-10

**Authors:** Jens Keilwagen, Jan Grau, Ivan A. Paponov, Stefan Posch, Marc Strickert, Ivo Grosse

**Affiliations:** 1 Molecular Genetics, Leibniz Institute of Plant Genetics and Crop Plant Research (IPK), Gatersleben, Germany; 2 Institute of Computer Science, Martin Luther University Halle–Wittenberg, Halle/Saale, Germany; 3 Institute of Biology II/Botany, Faculty of Biology, Albert–Ludwigs–University Freiburg, Freiburg, Germany; 4 Centre for Biological Signalling Studies (BIOSS), Albert–Ludwigs–University of Freiburg, Freiburg, Germany; Columbia University, United States of America

## Abstract

Transcription factors are a main component of gene regulation as they activate or repress gene expression by binding to specific binding sites in promoters. The de-novo discovery of transcription factor binding sites in target regions obtained by wet-lab experiments is a challenging problem in computational biology, which has not been fully solved yet. Here, we present a de-novo motif discovery tool called *Dispom* for finding differentially abundant transcription factor binding sites that models existing positional preferences of binding sites and adjusts the length of the motif in the learning process. Evaluating Dispom, we find that its prediction performance is superior to existing tools for de-novo motif discovery for 18 benchmark data sets with planted binding sites, and for a metazoan compendium based on experimental data from micro-array, ChIP-chip, ChIP-DSL, and DamID as well as Gene Ontology data. Finally, we apply Dispom to find binding sites differentially abundant in promoters of auxin-responsive genes extracted from *Arabidopsis thaliana* microarray data, and we find a motif that can be interpreted as a refined auxin responsive element predominately positioned in the 250-bp region upstream of the transcription start site. Using an independent data set of auxin-responsive genes, we find in genome-wide predictions that the refined motif is more specific for auxin-responsive genes than the canonical auxin-responsive element. In general, Dispom can be used to find differentially abundant motifs in sequences of any origin. However, the positional distribution learned by Dispom is especially beneficial if all sequences are aligned to some anchor point like the transcription start site in case of promoter sequences. We demonstrate that the combination of searching for differentially abundant motifs and inferring a position distribution from the data is beneficial for de-novo motif discovery. Hence, we make the tool freely available as a component of the open-source Java framework Jstacs and as a stand-alone application at http://www.jstacs.de/index.php/Dispom.

## Author Summary

Binding of transcription factors to promoters of genes, and subsequent enhancement or repression of transcription, is one of the main steps of transcriptional gene regulation. Direct or indirect wet-lab experiments allow the identification of approximate regions potentially bound or regulated by a transcription factor. Subsequently, de-novo motif discovery tools can be used for detecting the precise positions of binding sites. Many traditional tools focus on motifs over-represented in the target regions, which often turn out to be similarly over-represented in the entire genome. In contrast, several recent tools focus on differentially abundant motifs in target regions compared to a control set. As binding sites are often located at some preferred distance to the transcription start site, it is favorable to include this information into de-novo motif discovery. Here, we present Dispom a novel approach for learning differentially abundant motifs and their positional preferences simultaneously, which predicts binding sites with increased accuracy compared to many popular de-novo motif discovery tools. When applying Dispom to promoters of auxin-responsive genes of Arabidopsis thaliana, we find a binding motif slightly different from the canonical auxin-response element, which exhibits a strong positional preference and which is considerably more specific to auxin-responsive genes.

## Introduction

Gene regulation is a complex process controlled by many influential components such as the binding of proteins to DNA or the binding of miRNAs to mRNA, RNA editing, splicing of pre-mRNA, mRNA degradation, or post-translational modification. One of the fundamental regulatory steps is the binding of transcription factors (TFs) to the promoters of their target genes. TFs influence the initiation of transcription, which in turn affects many subsequent regulatory processes. TFs bind to their binding sites (BSs) via a DNA binding domain, and one challenge in computational biology is the identification of transcription factor binding sites (TFBSs) in the promoters of target genes.

Target regions of TFs can be obtained by a combination of different wet-lab experiments including electrophoretic mobility shift assays (EMSA) [1], DNAse footprinting [2], ELISA [3], [4], ChIP-chip [5], [6], ChIP-seq [7], or expression profiling [8]. However, the regions identified by these methods are large and not limited to TFBSs solely, so de-novo motif discovery tools are typically used for predicting putative TFBSs. These tools take a set of target promoters with unknown binding motif and unknown BSs as input and predict putative binding motifs and the corresponding putative BSs simultaneously.

A wealth of de-novo motif discovery tools has been developed over the last decades including, for example, Gibbs Sampler [9]–[11], MEME [12], Weeder [13], Improbizer [14], DME [15], DEME [16], or A-GLAM [17]. These tools differ by the learning principle employed to infer the model parameters and by their capability of learning the position distribution of the BSs from the data.

Many de-novo motif discovery tools including Gibbs Sampler [9]–[11], MEME [12], Weeder [13], Improbizer [14], and A-GLAM [17] use generative learning principles for discovering statistically over-represented motifs from a set of target promoters, i.e. motifs with the highest abundance in the target promoters. However, the discovered motifs often turn out to be similarly over-represented in the rest of the genome, diminishing the specificity of these motifs for the target promoters. In order to overcome this limitation, de-novo motif discovery tools using discriminative learning principles such as DME [15] and DEME [16] have been developed during the last years. These tools utilize an additional control data set expected to contain no or only few BSs of the motif of interest for discovering differentially abundant motifs, i.e. motifs with a high abundance in the set of target promoters and a lower abundance in the control data set.

Many de-novo motif discovery tools including Gibbs Sampler [9]–[11], MEME [12], Weeder [13], DME [15] and DEME [16] use a fixed position distribution, chosen to be a uniform distribution in most cases. Motivated by the observation that TFBSs often occur not uniformly distributed along the promoters [10], [18], [19], tools such as Improbizer [14] and A-GLAM [17] have been developed that are capable of learning the positional distribution from the data.

In Table 1, we categorize the above-mentioned tools according to their capability of (i) finding differentially abundant motifs and (ii) learning the position distribution from the data. None of these tools works perfectly [20], [21], but typically de-novo motif discovery tools utilizing a discriminative learning principle outperform those utilizing a generative learning principle [22], and de-novo motif discovery tools capable of learning the positional preference of TFBSs typically outperform those with a fixed distribution [17]. No algorithm has been developed that combines both features. Here, we introduce Dispom, a discriminative de-novo position distribution motif discovery tool that is capable of modeling the positional preference of TFBSs. Although we focus on the application of Dispom to the de-novo discovery of motifs of TFs in promoter sequences, Dispom may also be used for the discovery of differentially abundant motifs of other origin such as enhancers, silencers, insulators, or miRNA target sites.

**Table 1:** Overview of de-novo motif discovery tools.

	fixed	learned from data
**generative**	Gibbs Sampler, MEME, Weeder	Improbizer, A-GLAM
**discriminative**	DME, DEME	*Dispom*

Rows indicate the learning principle, and columns indicate if the position distribution can be learned from the data. Weeder uses a consensus-based representation of the motif, while the other tools use probabilistic models. None of the existing tools is capable of searching for differentially abundant BSs and learning the positional distribution simultaneously, and developing such a tool is the goal of this work. As this tool is capable of modeling the positional preference of TFBSs using a discriminative learning principle, we call it Dispom, a tool for discriminative de-novo position distribution and motif discovery.

Similar to other discriminative tools such as DEME or DME, Dispom utilizes a control data set assumed to contain no or few BSs of interest in addition to the target data set. And similar to Improbizer and A-GLAM, Dispom learns the distribution of binding positions from the data simultaneously with the parameters of the motif model. In addition, Dispom uses a heuristic during parameter learning for adapting the length of the binding motif, which is often unknown in advance, and for compensating phase shifts [9], which frequently occur in many de-novo motif discovery tools.

The remainder of this paper is structured as follows. In the section *Methods*, we describe Dispom and the data used in the subsequent case studies. In section *Results*, we compare the performance of Dispom based on the motif and on the BS level to that of commonly used de-novo motif discovery tools. For the motif level, we use the metazoan compendium proposed by Linhart et al. [23], while for the BS level we use 18 benchmark data sets with planted BSs investigating whether the tools are capable of finding motifs with and without positional preference. Finally, we apply Dispom to a data set of promoters of auxin-responsive genes in a cell suspension culture of *Arabidopsis thaliana*. We compare the motif found by Dispom with the canonical auxin-responsive element and test how specific these motifs are at predicting auxin-responsive genes for an independent data set.

## Materials and Methods

In this section we describe Dispom including the probabilistic model, the parameter learning principle, and a heuristic for avoiding phase shifts and for the inference of the motif length. Subsequently, we explain how we compare the performance of de-novo motif discovery tools, and we describe the data sets used in the case studies.

### Dispom – Model

Denote a DNA sequence of length 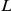 by 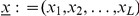, the nucleotide at position 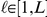 by 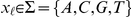, the subsequence from position 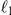 to 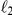 by 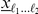, and the reverse complement of 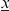 by 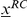. Dispom is based on the *Zero or One Occurrence Per Sequence* (ZOOPS) model used in many de-novo motif discovery tools [12], [14], [16], [17]. The ZOOPS model uses two hidden variables:

- The variable 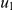 handles the possibility that a sequence does not contain a BS. 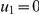 denotes the case that the sequence contains no BS, and 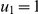 denotes the case that the sequence contains exactly one BS. If the sequence contains one BS, it can be located at different positions.
- If 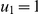, the variable 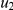 denotes the start position of a BS in the sequence.

Based on any *motif model* 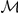 with motif length 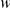, any *start position distribution* 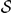, and any *flanking sequence model* 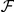, we obtain the likelihood for sequence 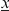 given parameters 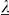


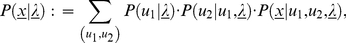
(1)where the sum runs over all possible combination of values of 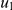 and 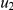, and 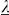 denotes the vector of model parameters. The probability 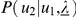 is defined as
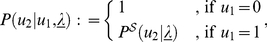
(2)where 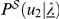 denotes the probability of 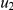 using the start position distribution 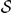. If the sequence 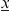 contains no BS, i.e., if 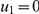, it is assumed that 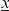 is generated by 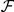


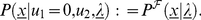
(3a)If the sequence 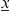 contains a BS, then it is assumed that the nucleotides upstream and downstream of the BS are generated by 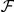, while the BS is generated by 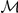. This yields
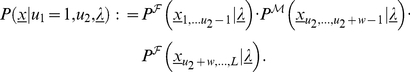
(3b)Similar to other tools, Dispom uses a position weight matrix as motif model 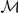 for both DNA strands and a homogeneous Markov model of order 0 as flanking sequence model 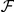.

In contrast to other tools, Dispom utilizes a mixture of a skew normal and a uniform distribution as position model 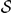. The choice is motivated by the observation that a Gaussian distribution decays quite rapidly, and hence, BSs further apart from the mean of the Gaussian are often overlooked. Similarly, the choice of the skew normal instead of a Gaussian distribution is inspired by the expectation that if the mean of the Gaussian is close to the transcription start site (TSS) there might be a skew of the distribution. Further details about the model can be found in Text S1.

For predicting BSs in a sequence 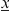, we compute the probability
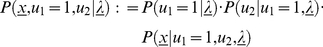
(4)for each possible position 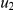 of 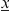. We also compute these probabilities for each possible position in each sequence of the control data set yielding a background distribution of probabilities. We define the 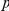-value of position 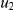 being erroneously predicted as a BS as the fraction of the probabilities that exceed the probability at position 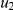 according to the background distribution. We finally define a threshold 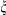 on the 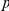-values, and predict all positions 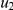 of a sequence with 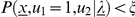 as starting positions of a BS.

### Dispom – Learning parameters

The goal of de-novo motif discovery is to infer proper parameters of the motif model from a set of target regions and, in case of discriminative approach, an additional set of control regions. We use a labeled data set of 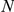 sequences where we denote the 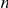-th sequence by 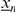 and its class label by 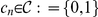. While tools like MEME and Improbizer use the generative maximum a-posterior (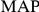) principle for learning the parameters based on a target data set, DME, DEME, and Dispom use a discriminative learning principle, and, hence, utilize an additional control data set. Dispom uses the maximum supervised posterior (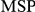) principle [24], [25], a discriminative Bayesian learning principle. The 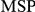 estimator of 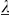 is defined by
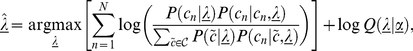
(5)where the first summand is the logarithm of the conditional likelihood, and second summand is the logarithm of the prior on the parameters 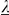 with hyper-parameters 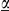. For the distribution 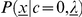 we choose the ZOOPS model described above, and for the distribution 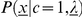 we follow the proposal of [16] and use a homogeneous Markov model of order 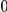. As prior, we choose a composite prior that utilizes Gaussian and Gamma distributions for the parameters of the position distribution and Dirichlet priors [26] for the sequence model. The hyper-parameters of these priors use mild assumptions, as for instance uniform pseudo-data for the motif model. Further details about the prior and the hyper-parameters can be found in Text S1.

We obtain estimates of the parameters of Dispom by numerical maximization [27] of Equation (5). Since the ZOOPS model implements a non-convex supervised posterior it may get trapped in local optima or saddle points. One prominent type of local optima are so-called phase shifts where the BSs are only covered by a part of the motif model. Besides starting Dispom multiple times, we implement a heuristic that helps reducing this problem and at the same time allows to adjust the motif length.

### Dispom – Phase shift and adjustment of motif length

Similar to other models, the ZOOPS model is prone to phase shifts. For this reason, we allow the motif model to be shifted, truncated, or expanded using a heuristic. The complete parameter learning including heuristic steps consists of the following four steps.

1. Maximize the model parameters 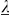 numerically using Equation (5).
2. Determine the number of *insignificant positions* on both sides of the motif model. *Insignificant positions* are contiguous positions at the borders of the motif model that can be removed without decreasing the number of promoters predicted to contain at least one BS by more than 20%.
3. Propose a *promising modification* of the motif model from the set of *insignificant positions*. A promising modification is a shift, a truncation, or an expansion of the motif model according to set of rules described in Text S1.
4. Compute the model parameters 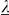 corresponding to the *promising modification* and restart the numerical optimization with these model parameters as initial values.

We ensure that these four steps do not lead to cycles by keeping a history of the performed steps. Text S1 contains further details about the heuristic.

### Dispom – Run time, limitations, and implementation

For non-convex functions, it is clear that the optimization algorithm can get trapped in local optima or saddle points. Hence, we start the optimization algorithm including the heuristic steps 50 times, and we choose those parameters 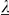 with the highest supervised posterior.

Due to these repeated starts of the numerical optimization, the runtime of Dispom is considerable. In Text S1, we present a comparison of the runtimes of Dispom and other tools for different data sets with varying numbers of sequences and with varying lengths. A single run of Dispom needs approximately the same run time as Weeder of up to several hours.

Conceptually, it is important to note that Dispom, like several other tools, is limited to model at most one BS per sequence, since it is based on the ZOOPS model. In Text S1, we investigate whether the assumptions of the ZOOPS model hamper Dispom in cases where these assumptions are not met. Second, Dispom only works on sequences of identical length, since the position distribution of the BSs is learned from the data. The length of the sequences can be defined by the user. We successfully tested different promoter lengths up to 1,200 bp, but typically the algorithm tends to work better for short sequences than for longer ones. Third, Dispom, like other discriminative de-novo motif discovery tools, requires a control data set for discriminative learning. If no specific control data set is available, one can randomly draw a control data set from the remaining promoters. Typically, we choose a control data set with at least as many sequences as in the target data set. For small target data sets, it is often useful to choose a larger control data set containing e.g. 1,000 sequences. Much larger control data sets typically yield only a marginal improvement of accuracy but increase the runtime unnecessarily. For the target data sets, we tested several sizes starting from a few dozen up to few thousand sequences. Typically, larger data sets yield better results than smaller data sets if for each sequence the probability of containing a BS is similar in both data sets.

Dispom is implemented in Jstacs (http://www.jstacs.de), an open-source and object-oriented Java framework for statistical analysis and classification of biological sequences. This enables users to apply and extend Dispom easily, e.g. by other sequence or position models, parameter initialization methods, learning principles, or heuristic steps.

### Comparison of de-novo motif discovery tools

Prediction performance of different de-novo motif discovery tools is usually compared using the *nucleotide recall* (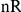) and the *nucleotide precision* (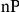), which are also referred to as *nucleotide sensitivity* and *nucleotide positive predictive value*, respectively [20]. Let the *true positives* 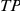 be the number of positions correctly predicted to be covered by BSs according to the annotation, let 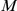 be the number of positions covered by BSs, and let 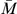 be the number of positions predicted to be covered by BSs. Then, 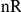 is defined as the fraction of correctly predicted nucleotides out of all nucleotides of all annotated BSs, 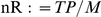, and 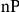 is defined as the fraction of correctly predicted nucleotides out of all nucleotides of all predicted BSs, 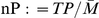.


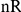
 and 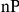 depend on parameters of the tools, such as the threshold 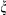. For this reason, the values of 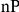 and 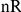 may be very different, and it is hard to compare the performance of different tools using only a single pair of 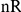 and 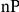. Typically, some tools have high values of 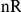 and low values of 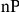, while other tools have low values of 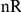 and high values of 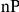, complicating a one-to-one comparison of their accuracy. Hence, we vary the threshold 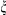, which is connected to the number of predictions, and obtain a series of pairs of 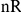 and 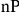 for each tool. Plotting these values of 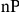 against 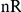 yields the *nucleotide precision recall curve*, which is more suitable for assessing imbalanced data sets than the commonly used ROC curve [28]–[31]. For the comparison, we use the predictions reported by the tools themselves. All of the tools provide some score or measure of significance together with their predictions, which we use to rank these prediction when computing 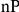 and 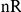 for different thresholds. Since, in contrast to Dispom, most tools operate with fixed internal thresholds resulting in a limited maximum 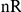, we can only obtain partial curves for these tools, which still provide more information than single pairs of 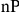 and 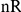 values.

### Data sets

In this subsection, we describe the data sets used for de-novo motif discovery in the results section. First, we briefly describe the metazoan compendium which is initially used for evaluating the performance of Amadeus [23]. Second, we describe the data sets used for comparing the prediction performance of Dispom with existing de-novo motif discovery tools. Third, we describe two data sets of auxin-responsive genes of *Arabidopsis thaliana* [32] that we use for applying Dispom to a real-life problem where the true motif and the true BSs are unknown.

#### Metazoan compendium from Amadeus

Several benchmark tests have been used for comparing different de-novo motif discovery tools over the last years. These comparisons are based on annotated BSs [17], [20] or on annotated binding motifs [23]. For an evaluation of de-novo motif discovery tools on the motif level, the metazoan compendium [23] is one of the most comprehensive benchmark data sets. It comprises 32 data sets for TFs and 10 data sets for miRNAs from human, mouse, *Caenorhabditis elegans*, and *Drosophila melanogaster*. Focusing on TFBSs in this paper, we choose data sets for the TFs that are based on data from micro-array, ChIP-chip, ChIP-DSL, and DamID experiments as well as data from Gene Ontology databases.

We follow the benchmark protocol used in [23] utilizing the normalized euclidean distance [33] and the TRANSFAC database [34]. The latest publicly available version (TRANSFAC v. 7.0 [35]) does not contain all matrices used by the benchmark protocol [23], which was compiled using the commercial TRANSFAC database 10.2, so we conduct the benchmark for the 24 data sets with at least one matrix available in TRANSFAC 7.0.

For each of the 24 target data sets, we create one control data set by randomly selecting promoters of the same species, and a second control data set by choosing promoters of the same species with similar GC-content as the promoters in the target data set. Each of the control data sets comprises at least 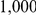 promoter sequences. If the target data set contains more than 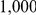 promoters, we select the same number of promoters for the control data set.

#### Benchmark data sets with implanted BSs

For an in-depth comparison of the performance of different de-novo motif discovery tools, a comparison based on BSs is essential. Data sets with annotated BSs have been used in [17], [20], but a simple analysis (Text S1) shows that motifs of length 8 to 10 bp can be found just by chance in randomly chosen promoters of the same size, stating that finding motifs of this length is often insignificant.

Hence, we choose to plant verified BSs into annotated promoters to obtain sufficiently large benchmark data sets (Dataset S1) with known BS positions as follows:

We download data sets of annotated BSs of seven TFs from the JASPAR database [36]. We choose those TFs with the greatest number of annotated BSs according to JASPAR, and we denote these data sets of BSs by their JASPAR-ID. These data sets cover TFs of mammals (three data sets: MA0048: 54 BSs; MA0052: 58 BSs; MA0077: 76 BSs), plants (three data sets: MA0001: 97 BSs; MA0005: 90 BSs; MA0054: 70 BSs), and insects (one data set: MA0015: 80 BSs).

We download promoters of the corresponding organisms for each of these seven data sets, and we extract for each promoter data set the upstream 500 bp relative to the TSS. We obtain promoters of *Arabidopsis thaliana* from TAIR (http://www.arabidopsis.org/), promoters of *Homo sapiens* from the Human Promoter Database (http://zlab.bu.edu/~mfrith/HPD.html), and promoters of *Drosophila melanogaster* from the Eukaryotic Promoter Database (http://www.epd.isb-sib.ch/index.html). In case of data set MA0054 from *Petunia x hybrida*, we use promoters of *Arabidopsis thaliana*, since promoters for *Petunia x hybrida* are not available.

For each of the seven data sets of TFBSs and the corresponding promoters, we create one data set with implanted BSs by the following procedure described for the example of data set MA0001.

1. We randomly choose 138 promoters of *A. thaliana*. Randomly select 97 out of these 138 promoters, and we implant one of the 97 BSs of data set MA0001 into each of them, either on the forward strand or the reverse complementary strand, using a uniform positional distribution. The number of promoters is chosen such that 70% of them have exactly one implanted BS.
2. In perfect analogy, we create an additional data set of exactly the same size by replacing the uniform positional distribution by a Gaussian distribution. We draw the mean and the standard deviation of the Gaussian distribution uniformly from the intervals 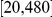 and 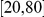, respectively. We choose an interval of 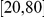 for the standard deviation to obtain a Gaussian distribution that substantially deviates from the uniform distribution.
3. In addition to these two target data sets, we create a control data set of exactly the same size by randomly choosing another 138 promoters of *A. thaliana* without implanting any BS. We combine this control data set with each of the two target data sets, yielding two pairs of benchmark data sets for TF MA0001.

We repeat this procedure for the remaining six TFs, yielding 14 pairs of benchmark data sets in total. Table 2 shows the number of implanted BSs and promoters for each of these data sets.

**Table 2:** Benchmark data sets.

motif ID	organism	number of BSs	number of target and control sequences each
MA0001	*A. thaliana*	97	138
MA0005	*A. thaliana*	90	128
MA0015	*D. melanogaster*	80	114
MA0048	*H. sapiens*	54	77
MA0052	*H. sapiens*	58	82
MA0054	*A. thaliana*	70	100
MA0077	*H. sapiens*	76	108

Rows indicate motifs and, hence, pairs of data sets for uniform and Gaussian distribution. Column one contains the motif ID, column two contains the organism, which is used for promoter extracting, column three contains the number of BSs stored in JASPAR, and column four contains the number of target and control sequences each.

We build four additional pairs of benchmark data sets containing a decoy motif in both the target *and* control data set as follows. We choose the two target data sets and the control data set of MA0048, and we plant a randomly chosen BS of MA0052 into each of the 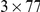 promoters, either on the forward strand or the reverse complementary strand, using a uniform positional distribution. We repeat this procedure in perfect analogy using a Gaussian positional distribution.

We denote the nine out of 18 pairs of data sets with BSs implanted by Gaussian distributions as *Gaussian data sets*, and we denote the remaining nine pairs of data sets as *uniform data sets*.

For the assessment of the nucleotide precision recall curves, we use the implanted BS positions and the BS lengths according to the annotation of JASPAR except for border positions with an information content of less than 0.25 bit in the sequence logo of the true motif, and we refer to these lengths as *correct motif lengths* in the following.

#### Data sets of auxin-responsive promoters

We use expression data of *Arabidopsis thaliana* from a cell suspension culture, because it is ideal for studying transcriptional responses to different stimuli due to its uniformity and homogeneity. The plant hormone auxin plays a critical role in virtually all aspects of plant growth and development and regulates the transcription of many genes [37]. Direct target genes of auxin response are known to be regulated quickly, so we select genes with a two-fold increase in gene expression after a short exposure time of 15, 30, or 60 minutes in the cell suspension culture [32]. As an independent set of genes, we select genes up-regulated in seedlings within the same time interval of 60 minutes after treatment [32] and the same threshold. We use the cell suspension data set containing 48 promoters as target data set, and we randomly select 1,000 promoters from the set of all remaining genes on the Affymetrix ATH1 microarray chip as control data set. For testing Dispom, we use the promoters of the seedling data set and of all remaining genes not used during training yielding 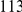 promoters and 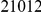 promoters, respectively. For all data sets, we use the promoter region from −500 bp to −1 bp relative to the TSS (Dataset S2).

## Results/Discussion

In this section, we first evaluate the performance of Dispom on the motif level using the metazoan compendium. Second, we compare the performance of the seven de-novo discovery tools A-GLAM, DEME, DME, Gibbs Sampler, Improbizer, MEME, and Weeder with that of Dispom on the BS level utilizing 18 benchmark data sets containing experimentally verified BSs. Finally, we apply Dispom to a situation where neither the motifs nor the true BSs are known. Specifically, we apply Dispom to promoters of genes up-regulated by auxin in a cell suspension culture of *Arabidopsis thaliana*, we compare the motif found by Dispom with the canonical auxin responsive element, and we investigate if the motif is also differentially abundant in the seedling data set compared to all remaining promoters.

### Evaluating Dispom on the motif level

We evaluate the performance of Dispom on the motif level for the 24 data sets of the metazoan compendium with at least one matrix available in TRANSFAC 7.0. To allow for an evaluation of Dispom using more recent versions of TRANSFAC, we make the motifs reported by Dispom for each of the 32 TFBS data sets of the metazoan compendium available at http://www.jstacs.de/index.php/Dispom.

In the original benchmark study [23], the performance of six tools, namely AlignACE, MEME, YMF, Trawler, Weeder, and Amadeus, is compared on the data sets of the metazoan compendium. Each tool is allowed to report two motifs of length 10 and two additional motifs of length 8. Out of these four motifs, the motif with the smallest normalized euclidean distance [33] is chosen to assess the performance of a tool [23]. The results achieved by the six tools with this procedure are available at http://acgt.cs.tau.ac.il/amadeus/suppl/results_metazoan.html, and we use the reported accuracies in the following comparison.

Since Dispom is capable of learning the length of the motif from the input data, we allow Dispom to report two different motifs of learned lengths as opposed to the four motifs considered for the other tools. We obtain the two motifs reported by Dispom for the two different types of control data sets described in subsection *Data sets*.

**Figure 1:**
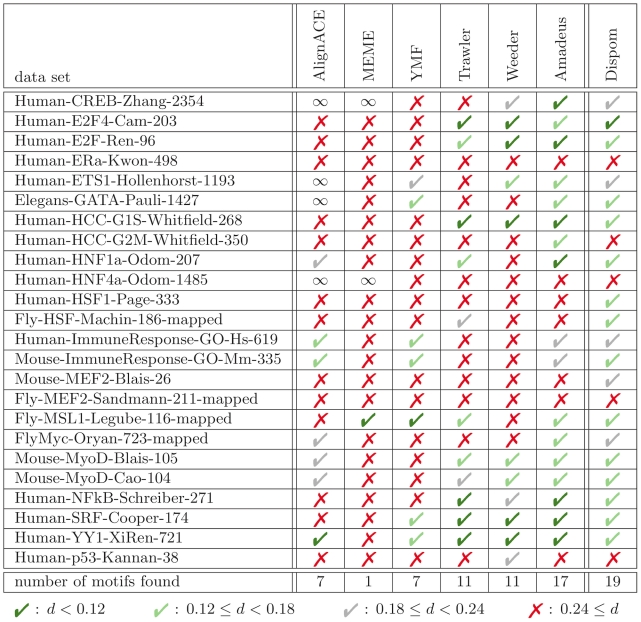
Comparison of de-novo motif discovery tools on the metazoan compendium. Each column of the table presents the results for one motif discovery tool, and each column corresponds to one data set of the metazoan compendium. We indicate by a red cross that a motif is not found, and we indicate by a checkmark, that a motif is found by a specific tool. The color of the checkmarks represents the accuracy of the motif discovered as measured by the normalized euclidean distance 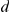, and we use the thresholds on the normalized euclidean distance as proposed by Linhart et al. [23]. The 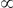 symbol marks long execution times (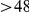
h) that were aborted in [23]. In the last row of the table, we report the total number of motifs discovered by each of the tools.

In Figure 1, we present the results of this comparison. We find that Dispom discovers the correct motif for 19 of the 24 data sets, whereas Amadeus correctly discovers 17 motifs, Weeder and Trawler discover 11 motifs, YMF and AlignACE discover 7 motifs, and MEME discovers 1 motif. While most of the motifs are discovered by at least three of the tools including Dispom, there are the following notable exceptions. For the data sets “Human-ERa-Kwon-498”, “Human-HNF4a-Odom-1485”, and “Fly-MEF2-Sandmann-211-mapped”, none of the tools considered is capable of discovering the correct motif, which demonstrates the importance of developing improved algorithms for de-novo motif discovery. For the data set “Human-HCC-G2M-Whitfield-350”, Amadeus is the only tool that finds the correct motif, and the correct motif of “Human-p53-Kannan-38” is found only by Weeder. Finally, in two cases, namely “Human-HSF1-Page-333” and “Mouse-MEF2-Blais-26”, Dispom is the only tool that finds the correct motif.

Considering the accuracy of the motifs reported by Dispom as measured by the normalized euclidean distance [33], we find a greater distance compared to other tools for some of the data sets. One explanation for this observation might be that for most of the data sets not all matrices that were used in the original benchmark [23] are available in TRANSFAC 7.0.

Summarizing these results, we may state that Dispom performs at least comparable to the best of the existing approaches on the metazoan compendium. Since Dispom is the only tools that finds the correct motif for the data sets “Human-HSF1-Page-333” and “Mouse-MEF2-Blais-26”, we may conclude that Dispom might be a valuable tools for discovering new motifs in data sets for which other tools failed in the past.

### Evaluating Dispom on the BS level

For testing the efficacy of Dispom, we compare it with commonly used available methods on the same data sets. First, we consider three different aspects of de-novo motif discovery for all tools. We consider the capability of de-novo motif discovery tools of

1. finding the correct BSs with unknown motif length,
2. recovering a non-uniform position distribution of the BSs in the data sets, and
3. finding differentially abundant motifs in the presence of non-specific but over-represented motifs.

For each of these issues, we consider only one specific example, and we present the remaining results in Figures S1, S2, S3, and S4. Finally, we provide an overview of the performance of the different de-novo motif discovery tools applied to each benchmark data set.

We run all of the programs using default parameters with the following exceptions: if available and not the default, we use switches for searching on both strands, for enabling a position distribution, and for using the ZOOPS model instead of the OOPS model. We start each of the programs **–** including Dispom **–** once specifying the correct length of the motif and once with switches for the automatic adaption of motif length. If such a switch is not available, we set the length of the motif to 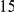. A list of the calls for all programs is given in Text S1.

#### Unknown motif length

First, we consider the aspect of finding the correct motif if the motif length is unknown. In many cases, when de-novo motif discovery tools are used, the user only has a rough idea of the motif length. Hence, the user must test all potential motif lengths and decide which result is of interest, or the tool allows to infer the motif length on its own.

**Figure 2:**
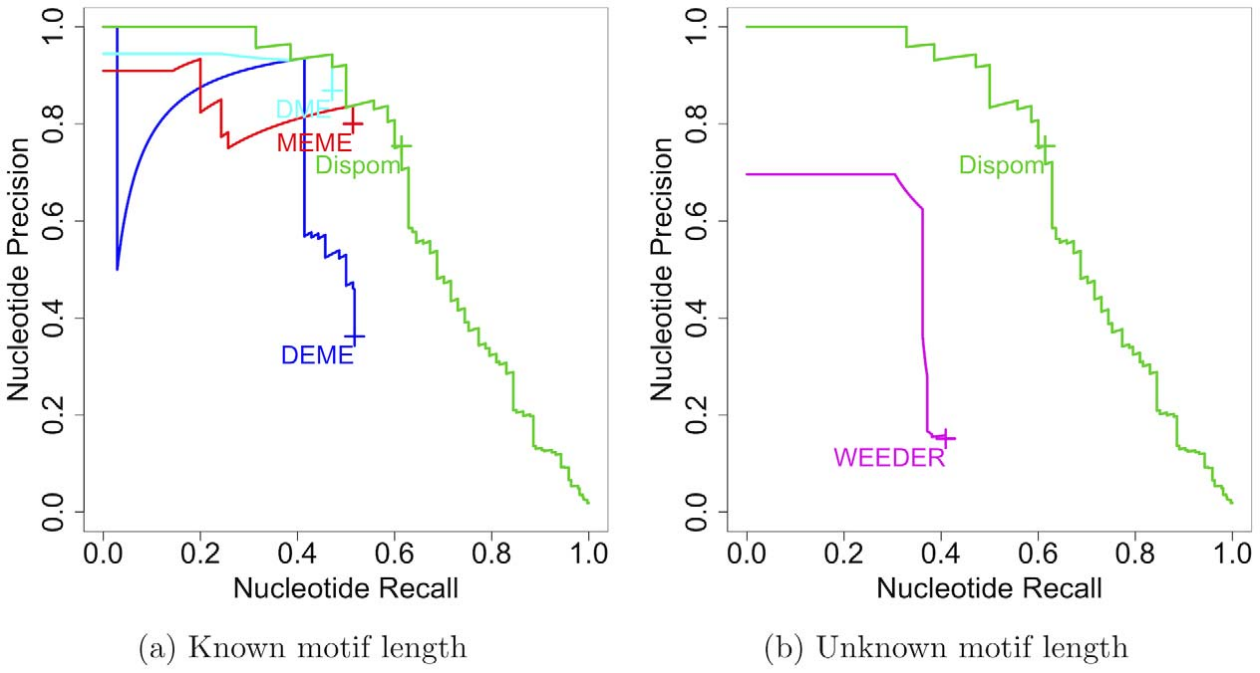
Comparison of nucleotide precision recall curves for known and unknown motif length. Figure 2a) shows the nucleotide precision recall curves for the de-novo motif discovery tools provided with the correct motif length, and Figure 2b) shows the nucleotide precision recall curves for the de-novo motif discovery tools when the correct motif length is not provided but must be learned by the tools. For reasons of visual clarity, we do not plot the partial nucleotide precision recall curves of those tools with 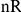 and 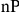 below 0.1 for all available thresholds. These curves would be located in the lower left corner of both subfigures.

Here, we study the results for different de-novo motif discovery tools for the target data set containing BSs of MA0054 with a Gaussian distribution, which is described in detail in section “Benchmark data sets with implanted BSs” of “Materials and Methods.” In the first experiment, we start all tools with the correct motif length. In the second experiment, we start all tools with an initial length of 15 bp, and allow to adjust the motif length if supported by the tool. In Figure 2, we show the results for both cases.

For known correct motif length, we find that DEME, DME, MEME, and Dispom find the implanted motif to a certain degree, i.e. it provides a 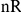 and 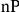 above 0.1 for at least one available threshold, showing that these four tools are capable of finding the implanted BSs. Among these four tools, Dispom performs best, and DEME, DME, and MEME perform comparably well. However, in case of unknown motif length, we find that DEME, DME, and MEME are not capable of finding the correct motif. While DEME and DME are not capable of adjusting the motif length, MEME allows searching the motif for a range of possible motif lengths. Nevertheless, all three tools fail to find the motif if the correct motif length is not provided.

In contrast to these findings, Weeder and Dispom are capable of finding the correct motif. Weeder is capable of finding the motif to a certain degree, although it is not capable of finding the motif for the known motif length. Scrutinizing the motif found by Weeder, we find that it is shorter than the true motif (Figure S4). In contrast, we find that the performance of Dispom is very similar to the case of known motif length indicating that Dispom is capable of finding the correct motif including the motif length.

Based on these case studies, we can state that knowing the correct motif length improves de-novo motif discovery. However, in many real-life applications, the correct motif length is unknown, and many de-novo motif discovery tools suffer in this situation. Dispom with its heuristic for truncating and expanding the motif is capable of learning the correct motif length from the data, and so, outperforms other de-novo motif discovery tools.

#### Non-uniform position distribution

**Figure 3:**
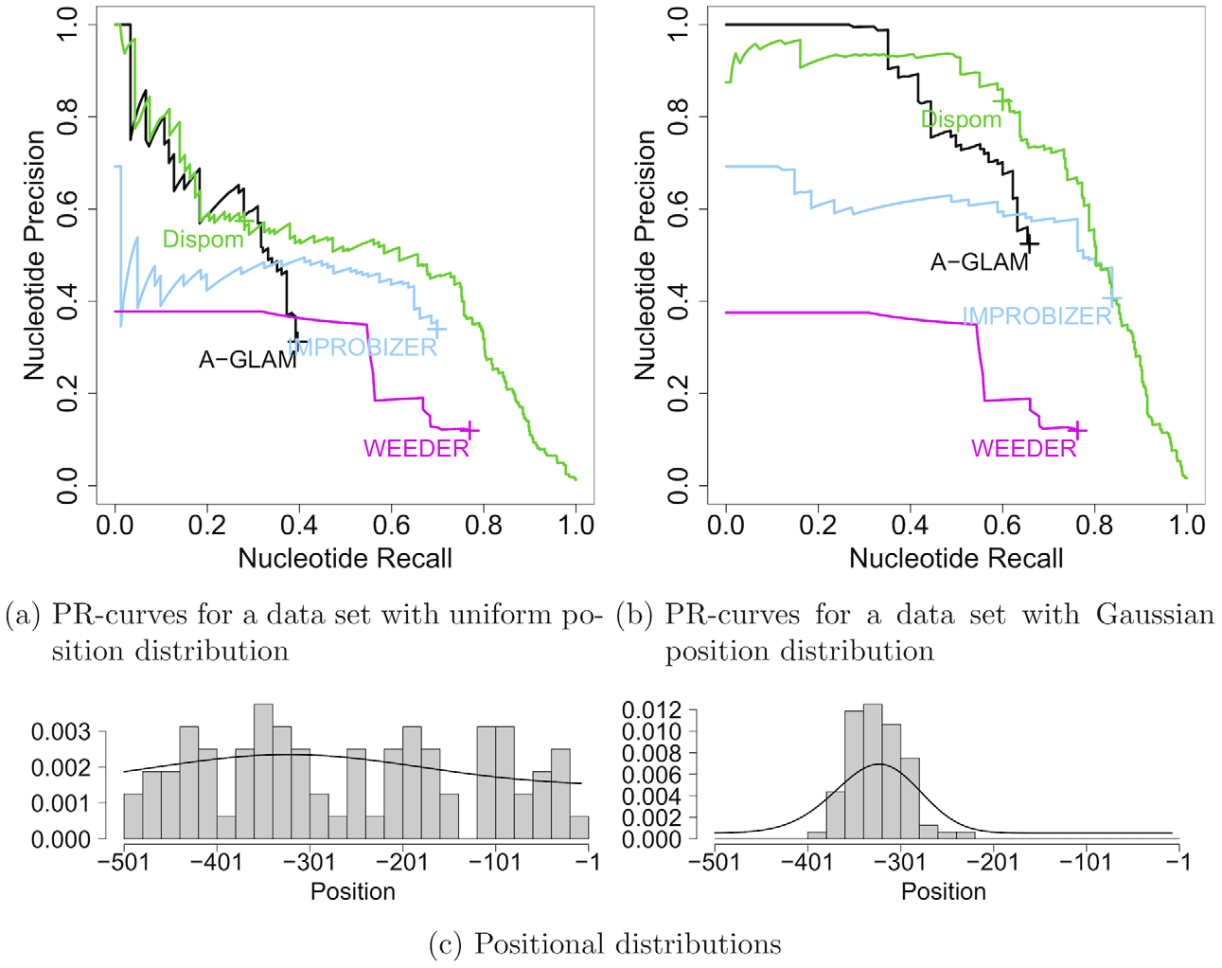
Comparison of nucleotide precision recall curves for uniform and Gaussian position distribution. Figure 3a) shows the nucleotide precision recall curves for the de-novo motif discovery tools on the data set with uniformly placed MA0015 BSs, and Figure 3b) shows the nucleotide precision recall curves for the de-novo motif discovery tools on the data set with Gaussian distributed MA0015 BSs. Figure 3c) shows for both data sets the real distributions as histograms of start positions of the implanted BSs and the position distributions learned by Dispom. For reasons of visual clarity, we do not plot results located in the lower left corners of subfigures a) and b) (cf. Figure 2).

Second, we consider the aspect of recovering a non-uniform position distribution of the BSs in the data set. In many cases, BSs are not uniformly distributed over the entire promoter but rather concentrated with a TF-specific position distribution. To simulate these findings, we use the data sets for MA0015 for which we compare the results of the Gaussian data set to those obtained for the uniform data set. Both data sets are described in detail in section “Benchmark data sets with implanted BSs” of “Materials and Methods.” Since both data sets consist of exactly the same BSs and the same promoters, and only differ in the position distribution used to implant the BSs, we are able to measure the effect of modeling a non-uniform position distribution. Figure 3 a) and b) show the nucleotide precision recall curves for both position distributions used for implanting the BSs.

For a uniform position distribution we observe that A-GLAM, Improbizer, Weeder, and Dispom find the correct motif. Turning to the case of a Gaussian position distribution, we observe that A-GLAM, Improbizer, and Dispom are able to utilize the positional preference of BSs to substantially improve their performance. In contrast to these findings, the performance of Weeder does not improve, because it does not model positional preference.

We scrutinize performance improvements by comparing the distribution used for implanting the BSs with the distribution learned by Dispom. In Figure 3 c), we show for both cases **–** the uniform and the Gaussian position distribution **–** a histogram for the start positions of the implanted BSs and the distribution learned by Dispom. We find that both distributions are in agreement in both cases, indicating that Dispom is capable of learning the position distribution from the data.

Based on these case studies, we can state that recovering the position distribution of the BSs from the data helps in de-novo motif discovery and the subsequent prediction of BSs. Since Dispom is able to learn peaked as well as uniform position distributions from the data, it can be used for in a wide range of applications.

#### Differentially abundant vs. over-represented motifs

Third, we consider the aspect of distinguishing between over-represented and differentially abundant motifs in the data set. Typically, promoters contain BSs of many different TFs. When applying de-novo motif discovery tools to such sequences, not all of these motifs are equally relevant. For instance, when comparing promoters of differentially and non-differentially expressed genes for a specific condition, we are typically interested in those motifs that differentially abundant in these sets of promoters and not in those motifs that are common to the promoters of both types of genes. Hence, it is beneficial for a de-novo motif discovery tool to distinguish between over-represented and relevant motifs.

**Figure 4:**
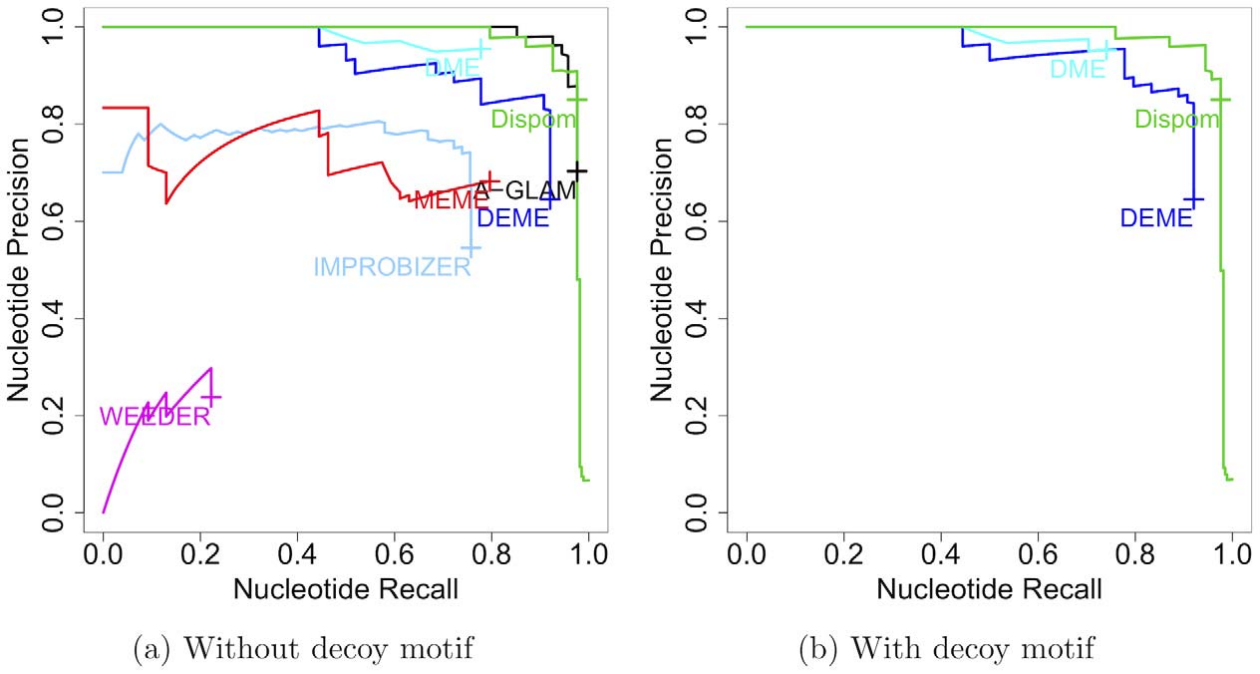
Comparison of nucleotide precision recall curves with and without decoy motif. Figure 4a) shows the nucleotide precision recall curves for the de-novo motif discovery tools on the data set without implanted decoy motif, and Figure 4b) shows the nucleotide precision recall curves for the de-novo motif discovery tools on the data set with implanted decoy motif MA0052. For both subfigures, we do not plot results located in the left lower corner for reasons of clarity (cf. Figure 2).

Here, we consider the target data set containing BSs of MA0048 with a Gaussian distribution, which is described in detail in section “Benchmark data sets with implanted BSs” of “Materials and Methods.” We compare the results for a data set with a uniformly implanted decoy motif (MA0052) to the same data set without implanted decoy motif. In Figure 4, we show the comparison of the nucleotide precision recall curves for known motif length. In case of no decoy motif, we observe that A-GLAM, DEME, DME, Improbizer, MEME, Weeder, and Dispom are capable of finding the correct motif. In a comparison, A-GLAM, DEME, DME, and Dispom perform best, Improbizer and MEME perform second best, and Weeder performs third best of these tools.

Considering the data set containing a decoy motif, we observe that A-GLAM, Improbizer, MEME, and Weeder, which are not designed for finding motifs that are differentially abundant in two data sets, are not capable of finding the correct motif. Characteristically, Improbizer, MEME, and Weeder find the unspecific decoy motif (Figure S3). In contrast, DEME, DME, and Dispom, which are specially designed for finding differentially abundant BSs, are capable of finding the correct motif.

Based on these case studies, we can state that discriminative de-novo motif discovery tools are capable of distinguishing between over-represented and differentially abundant motifs. This property is useful for finding motifs that help to discriminate between two data sets. The discriminative de-novo motif discovery tools DEME, DME, and Dispom are capable of finding the correct motif irrespective of the absence or presence of a decoy motif, so they perform similarly well in both cases.

#### Comprehensive comparison

**Figure 5:**
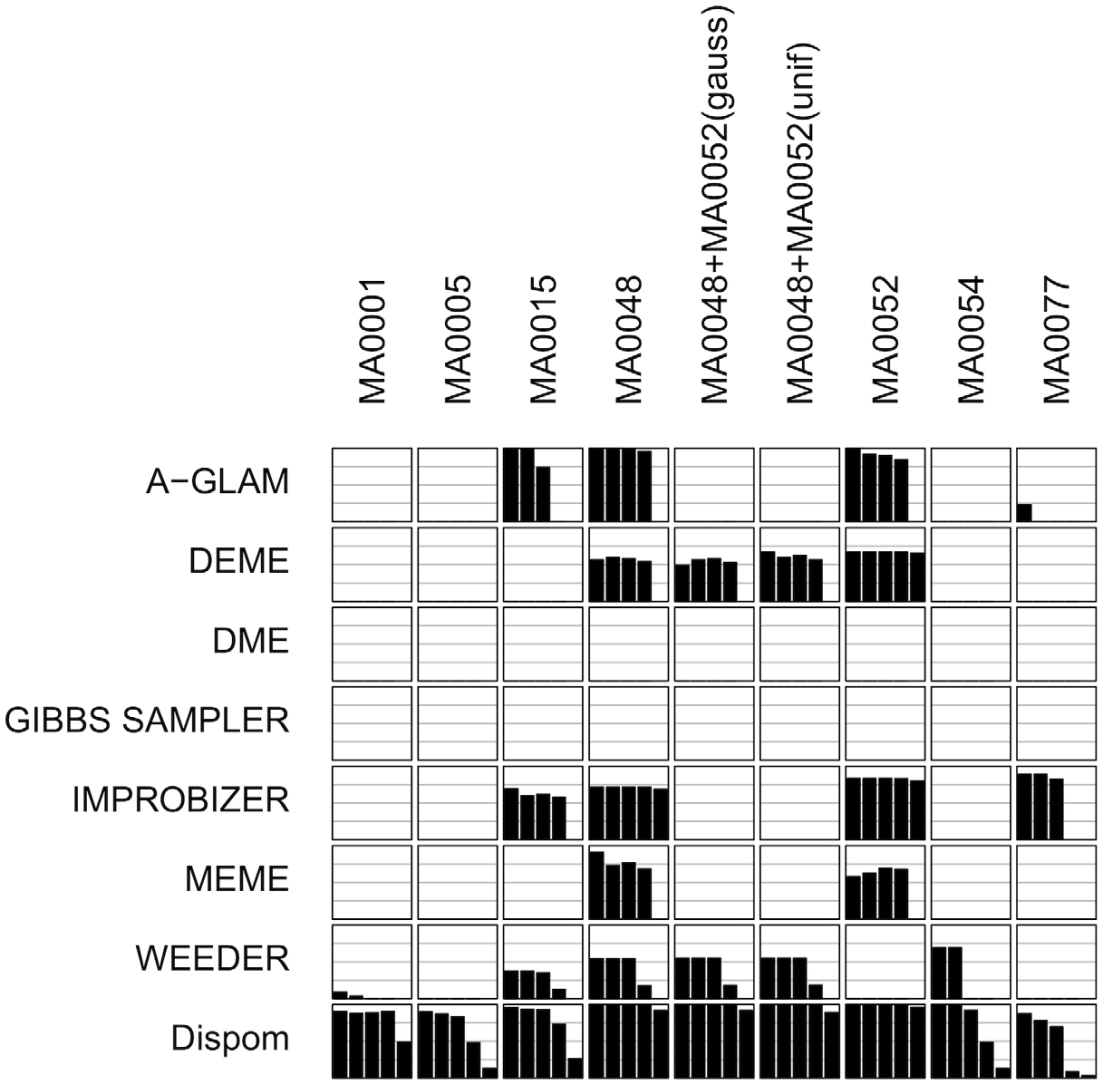
Overview of de-novo motif discovery results for Gaussian data sets and unknown motif length. Each column shows the results of one data set, and each row shows the results of one de-novo motif discovery tool. Each subfigure shows five bars that visualize the nucleotide precision for a nucleotide recall of 10%, 30%, 50%, 70%, and 90%, respectively, from left to right. Additionally, each subfigure contains gray horizontal lines for the nucleotide precision of 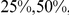 and 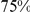.

After investigating three aspects of de-novo motif discovery in detail, we now compare all eight tools based on several data sets. To summarize this comparison, we show the nucleotide precision achieved for a nucleotide recall of 10%, 30%, 50%, 70%, and 90%. Based on the partial nucleotide precision recall curves for some tools, we may obtain missing values for some nucleotide recalls of some tools and some data sets, due to internal thresholds. In Figure 5, we consider the Gaussian data sets and unknown motif length. Complete and partial nucleotide precision recall curves as well as summaries similar to Figure 5 can be found in the Figures S1, S2, S3, and S4.

For an initial assessment, we first determine for each tool the number of data sets where not exclusively missing values are observed. We find that DME and Gibbs Sampler are unsuccessful in all data sets, while MEME is successful in two data sets, A-GLAM, DEME, and Improbizer in four data sets, Weeder in six data sets, and Dispom in all nine data sets. This initial assessment might be unfair for some tools, since it does not take into account the achieved values of the nucleotide precision. For example, A-GLAM and Improbizer often achieve very high nucleotide precisions, which is not considered in the initial assessment. Hence, we perform a second assessment in which we require a minimum nucleotide precision of 75%. We find that DEME, DME, Gibbs Sampler, and Weeder are unsuccessful in all data sets, while MEME is successful in one data set, Improbizer in two data sets, A-GLAM in three data sets, and Dispom in all nine data sets.

Considering the plant data sets, MA0001, MA0005, and MA0054, we find that most of the tools fail to find the correct motif while Dispom finds the motif in all three cases. Considering the results for the other data sets and for known motif length (Figures S1, S2, S3, and S4), we find similar results for unknown motif length on the uniform data sets and slightly better results for known motif length on both data sets. This indicates that the knowledge of the motif length has a decisive influence on the performance of many of the studied de-novo motif discovery tools. Especially DME, which performs poor in this case study (Figure 5), improves if the correct motif length is provided (Figure S3). Since Dispom is capable of adapting the motif length from the data, it outperforms the other tools.

### Applying Dispom to promoters of auxin-responsive genes

In the previous subsection, we compared the performance of Dispom and seven commonly used tools based on 18 data sets, suggesting that Dispom might be useful for finding differentially abundant BSs and their positional preference. In this subsection, we apply Dispom to promoters of auxin-responsive genes with the goal of finding putative TFBSs.

Auxin-responsive genes are regulated by a set of TFs commonly called auxin-responsive factors (ARF), which bind to auxin responsive elements (AuxREs) that occur in the promoters of those genes. The canonical AuxRE TGTCTC has been identified as a sequence specifically bound by ARF1 using gel mobility shift assays [38]. However, the ARF multi-gene family consists of 23 members [39], suggesting that AuxREs might differ for different members of ARFs. Indeed, subsequent analyses of 10 members of the ARF family indicate that only the first four nucleotides TGTC are essential for ARF-binding [40].

**Figure 6:**
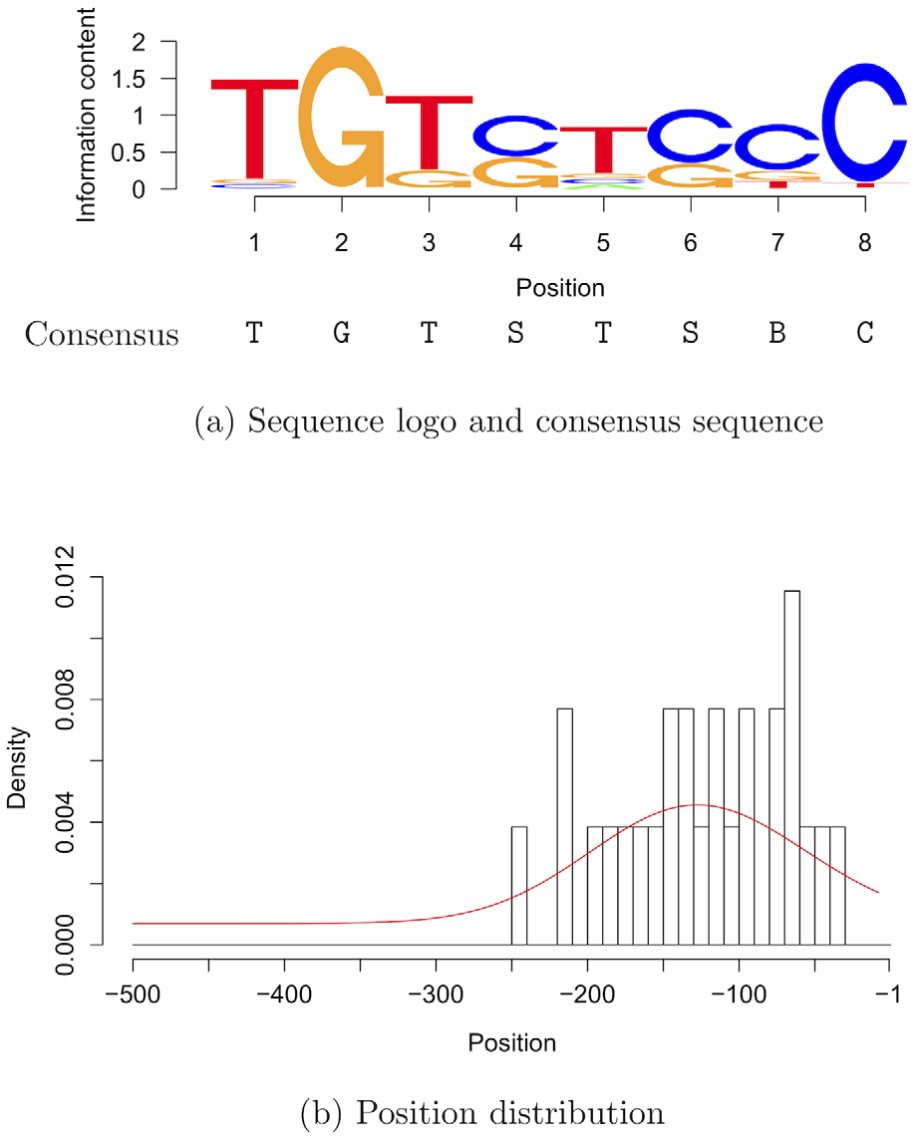
Auxin-dependent motif and position distribution found by Dispom. Figure 6a) shows the sequence logo obtained from the predictions of Dispom and the corresponding consensus sequence, where S stands for C or G, and B stands for C, G, or T. Figure 6b) shows a histogram of the predicted start positions and the position distribution learned by Dispom (red line).

Analyses of genome-wide expression data are based on the assumptions that co-expressed genes are regulated by the same TFs and the majority of their promoters contains BSs of these TFs. We use expression data sets for searching for a refined AuxRE. We apply Dispom to a set of promoters of genes up-regulated by the plant hormone auxin in *Arabidopsis thaliana* grown in a cell suspension culture [32]. Figure 6 visualizes the results of Dispom as a sequence logo [41] and the positional preference corresponding to this motif. We find a motif of length 8 bp predominately positioned in the 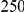-bp region upstream of the transcription start site. The core motif can be described as TGTSTSBC and can be interpreted as an elongated and modified version of the canonical AuxRE TGTCTC.

The presence of the canonical AuxRE TGTCTC in the promoters of a gene is often used as an indicator that this gene is auxin-responsive. For avoiding parameter overfitting, we use an independent test data set for evaluating the discriminative power of the found consensus sequence. We use the seedling data set described in the section Methods as target test data set, and we use the promoters of all remaining genes on the chip as control test data set. Interestingly, the restriction to the first four nucleotides TGTC, considered by some authors to be an improvement over the canonical ARF motif [40], decreases rather than increases the specificity. In Table 3, we summarize the results for the canonical AuxRE motif and the TGTSTSBC motif for the 500-bp upstream regions and the 250-bp upstream regions. For a more detailed analysis, we refer the reader to Table S1.

**Table 3:** Frequencies and significance for two auxin-dependent motif descriptions.

		seedling data set			control data set				
consensus	interval	match	no match	Sn	match	no match	FPR	F	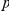 -value
**TGTCTC**	[−500,−1]	36	77	32%	4741	16271	23%	0.015	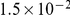
**TGTCTC**	[−250,−1]	26	87	23%	2564	18448	12%	0.019	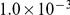
**TGTSTSBC**	[−500,−1]	26	87	23%	2305	18707	11%	0.021	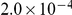
**TGTSTSBC**	[−250,−1]	21	92	19%	1252	19760	6%	0.030	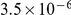

Each row provides the numbers for one consensus and interval combination. Column one and two contain the consensus and the interval. Column three to five contain the numbers for the seedling data set, where column three provides the number of promoters containing the consensus in the interval, column four provides the number of promoters that do not contain the consensus in the interval, and column five contains the recall (sensitivity, Sn) of the consensus in the specified interval. Likewise, column six to eight contain the numbers for the control data set, where FPR denotes the false positive rate of the consensus in the specified interval. Finally, column nine contains the F-measure (F) defined as the harmonic mean of precision and recall, and column ten contains the 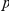-value obtained from Fisher's exact test using the confusion matrix based on columns three, four, six, and seven.

First, we compare the sensitivities and false positive rates of the different consensus sequences using the 500-bp region. We find (Table 3, lines 1 and 3) that the sensitivity decreases from 32% to 23% when replacing the canonical AuxRE by the refined motif TGTSTSBC. This decrease is clearly visible, but statistically non-significant, with a 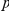-value of 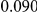 using the one-sided binomial proportion test. Turning to the false positive rate, we find that it decreases from 23% to 11% when replacing the canonical AuxRE by the refined motif TGTSTSBC. This decrease is highly significant with a 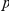-value of 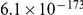 using the one-sided binomial proportion test. Hence, the refined motif is slightly less sensitive but significantly more specific than the canonical AuxRE.

Next, we compare the sensitivities and false positive rates for the canonical AuxRE in the 500-bp region and the refined motif TGTSTSBC in the 250-bp region. We find (Table 3, lines 1 and 4) that the sensitivity decreases from 32% to 19% when replacing the canonical AuxRE and the 500-bp region by the refined motif and the 250-bp region, yielding a 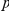-value of 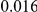 using the one-sided binomial proportion test. Turning to the false positive rate, we find that it decreases from 23% to 6%, yielding a 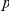-value below 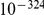. This very small 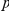-value states that replacing the canonical AuxRE by the refined motif and replacing the 500-bp region by the 250-bp region yields a highly significant decrease of the false positive rate corresponding to a highly significant increase of the specificity.

Finally, we assess the two consensus sequences and the two upstream regions using the F-measure and the 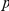-value of Fisher's exact test, which both consider the complete contingency table and combine sensitivity and false positive rate, for each of the four lines in Table 3. We find that combining the canonical motif TGTSTSBC and the 500-bp region yields an F-measure of 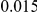, which is increased to 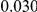 in case of the refined motif TGTSTSBC and the refined 250-bp region. This reflects the reduction of false predictions by a factor of 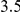 due to the refined motif and the refined upstream region detected by Dispom. In addition, we find the lowest 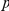-value of 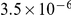 for the refined motif combined with the refined region. These observations illustrate the potential of combining discriminative de-novo motif discovery with the approach of simultaneously learning the positional distribution.

### Conclusions

Gene regulation and specifically the binding of TFs to their BSs is of fundamental interest in many areas of genome biology. A combination of experimental and computational methods are typically used for finding putative TFBSs. For computational approaches, two fundamental improvements have been proposed in the last years. On the one hand searching for differentially abundant motifs, and on the other hand learning a position distribution have been shown to be promising in several experiments separately. However, up to now there is no tool combining both improvements. We present Dispom, a new computational tool for the de-novo motif discovery that combines the capability of searching for differentially abundant BSs with the capability of learning the positional preference of the BSs. Dispom includes a heuristic for finding motifs of unknown length. We evaluate Dispom on benchmark data sets of the metazoan compendium and find that Dispom discovers two motifs that could not be found by any of the other tools considered. Additionally, we compare the performance of Dispom with seven commonly used de-novo motif discovery tools based on 18 data sets, and we find that Dispom outperforms these tools. Especially in cases where the correct motif length is not provided, the predictions of Dispom are substantially more accurate than those of traditional de-novo discovery tools indicating that the combination of discriminative learning, inferring a position distribution from the data, and utilizing a heuristic for finding the motif length is beneficial for de-novo motif discovery. Finally, we use Dispom on a set of auxin-responsive genes where the true motif is unknown. We find the motif TGTSTSBC, which can be interpreted as an refined AuxRE, predominantly located in the promoter region of 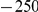 to 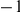. Both the refined motif as well as the refined promoter region lead to an improved discrimination of auxin-responsive and non-responsive genes on an independent genome-scale test data set. community as part of the open-source Java library Jstacs (http://www.jstacs.de), which allows an easy application, automation, and extension.

## Supporting Information

Dataset S1 Benchmark data sets.
(0.42 MB ZIP)

Dataset S2 Auxin data sets.
(0.20 MB ZIP)

Figure S1 Artificial data sets with uniform position distribution and known motif length: Nucleotide precision recall curves, sequences logos, and position distributions.
(3.66 MB PDF)

Figure S2 Artificial data sets with uniform position distribution and unknown motif length: Nucleotide precision recall curves, sequences logos, and position distributions.
(5.12 MB PDF)

Figure S3 Artificial data sets with Gaussian position distribution and known motif length: Nucleotide precision recall curves, sequences logos, and position distributions.
(3.65 MB PDF)

Figure S4 Artificial data sets with Gaussian position distribution and unknown motif length: Nucleotide precision recall curves, sequences logos, and position distributions.
(5.00 MB PDF)

Table S1 Binding site statistic for all genes of Arabidopsis thaliana. This file contains the number of BSs based on the 3 consensus sequences for all genes of Arabidopsis thaliana. The table includes the strand information and distinguishes between the promoter regions [−500,−1] and [−250,−1].
(7.01 MB XLS)

Text S1 This file contains the appendices of the manuscript including, for instance, additional information about the ZOOPS model, the prior and the hyper-parameters, the heuristic of Dispom, a simulation determining the length of motifs found in randomly drawn sets of promoters, a runtime comparison, the calls of the de-novo motif discovery tools, as well as a case study evaluating the restrictions based on the ZOOPS model.
(0.43 MB PDF)
