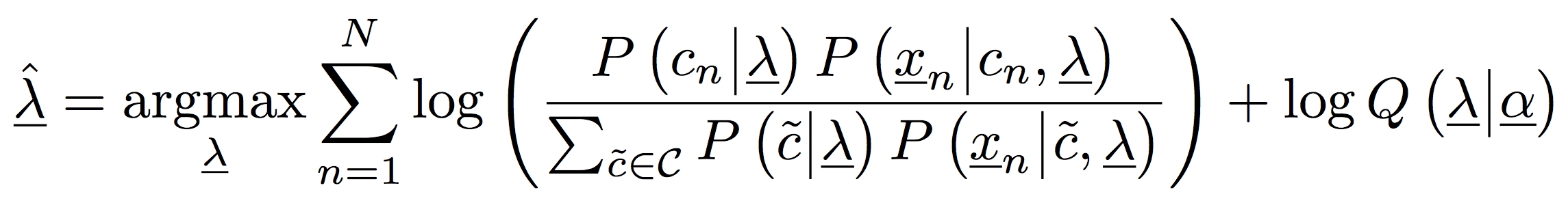# Correction: De-Novo Discovery of Differentially Abundant Transcription Factor Binding Sites Including Their Positional Preference

**DOI:** 10.1371/annotation/a0b541dc-472b-4076-a435-499ce9519335

**Published:** 2011-10-27

**Authors:** Jens Keilwagen, Jan Grau, Ivan A. Paponov, Stefan Posch, Marc Strickert, Ivo Grosse

In the Materials and Methods section, there was an error in the fifth equation of the section titled "Dispom - Learning parameters". 

Please view the complete, correct equation here: