# Supplementary material for: De-Novo Discovery of Differentially Abundant Transcription Factor Binding Sites Including Their Positional Preference
Source: PLoS Comput Biol. 2011 Feb 10;7(2):e1001070. doi: 10.1371/journal.pcbi.1001070 (PMC3037384; doi:10.1371/journal.pcbi.1001070)

Figure S1: All results for artificial data set (position distribution = unif; given length = true). For each data set, we show the nucleotide precison recal curve, the sequence logo obtained from each of the tools, and the position distribution learned by Dispom. Finally, we show a simplified overview of these results.

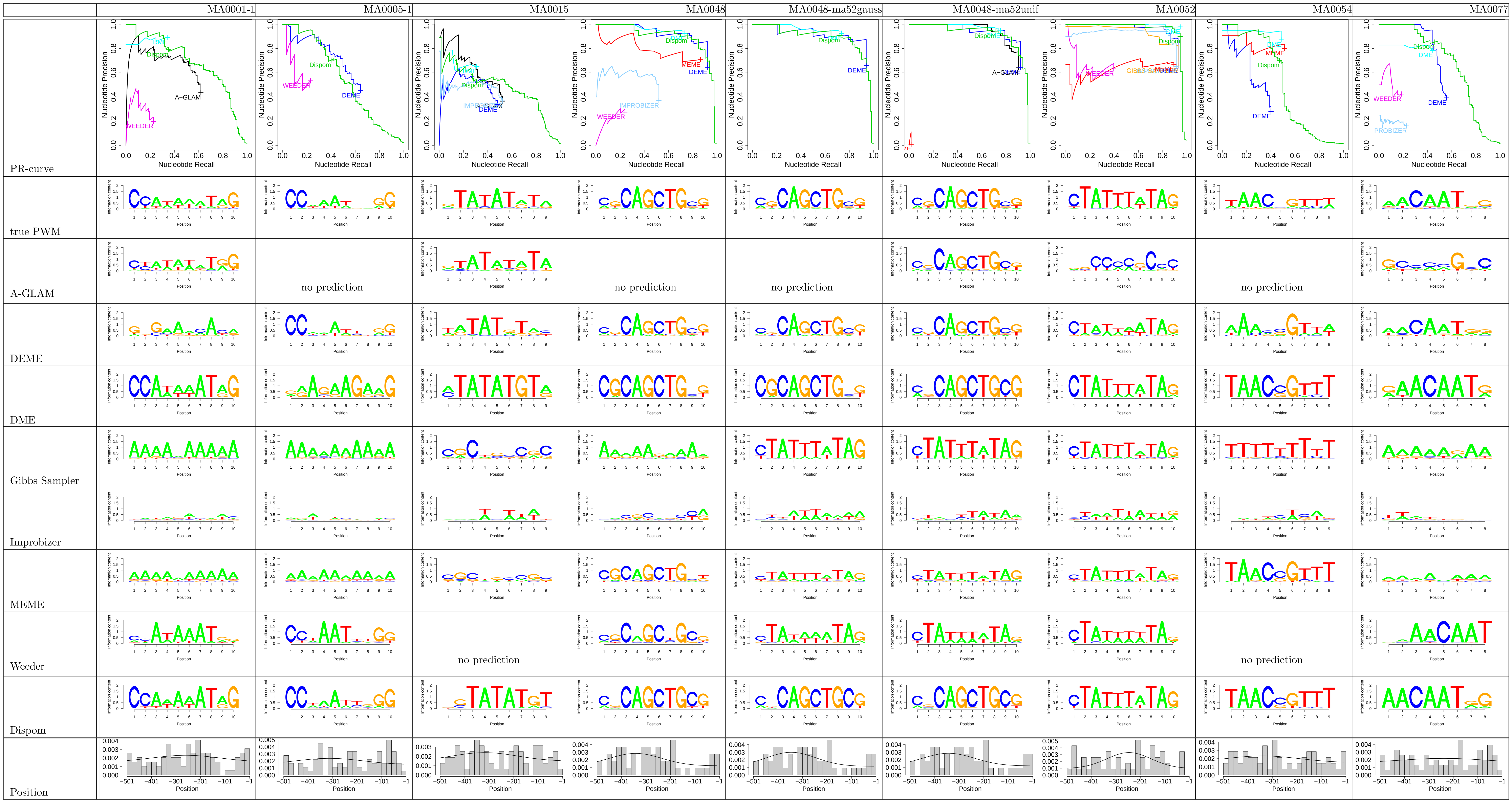

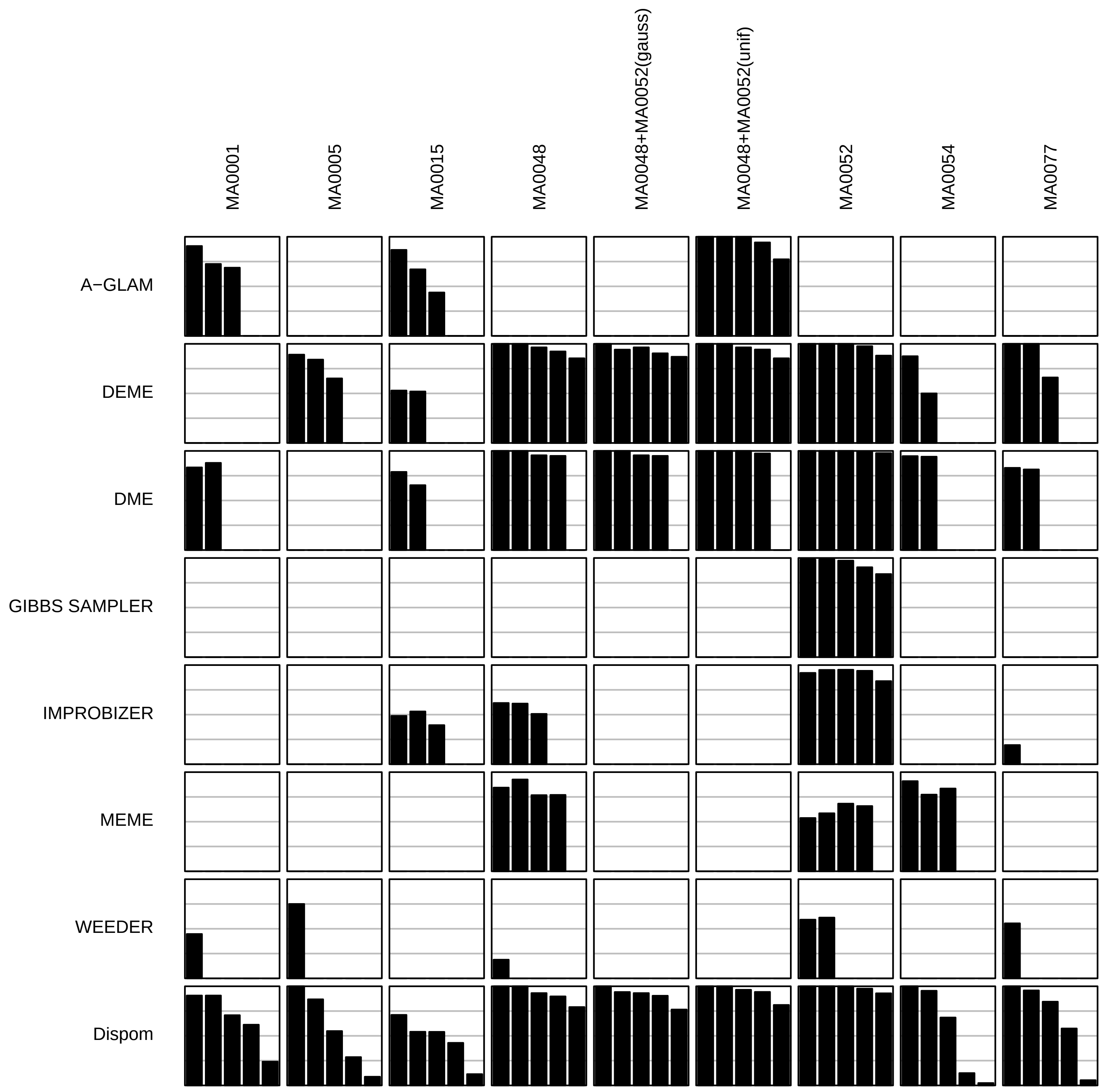

Supplement: Figure S1 — Artificial data sets with uniform position distribution and known motif length: Nucleotide precision recall curves, sequences logos, and position distributions. (3.66 MB PDF) [file pcbi.1001070.s003.pdf]
