# Supplementary material for: De-Novo Discovery of Differentially Abundant Transcription Factor Binding Sites Including Their Positional Preference
Source: PLoS Comput Biol. 2011 Feb 10;7(2):e1001070. doi: 10.1371/journal.pcbi.1001070 (PMC3037384; doi:10.1371/journal.pcbi.1001070)
Supplement: Text S1 — This file contains the appendices of the manuscript including, for instance, additional information about the ZOOPS model, the prior and the hyper-parameters, the heuristic of Dispom, a simulation determining the length of motifs found in randomly drawn sets of promoters, a runtime comparison, the calls of the de-novo motif discovery tools, as well as a case study evaluating the restrictions based on the ZOOPS model. (0.43 MB PDF) [file pcbi.1001070.s008.pdf]

Appendix A: Component models and parameterization

In this section, we define component models used in Dispom and their parameterization. We choose parameterizations that allow for an easy handling during numerical optimization, i.e. the parameters are unconstrained. Here, we describe the parameter vector $\underline{\lambda}$ more detailed. The vector is composed of the parameters $\underline{\lambda}^{(\mathcal{M})}$ of the motif model $\mathcal{M}$, the parameters $\underline{\lambda}^{(\mathcal{S})}$ of the start position distribution $\mathcal{S}$, and the parameters $\underline{\lambda}^{(\mathcal{F})}$ of the flanking sequence model $\mathcal{F}$ as well as the parameters $\underline{\lambda}_m$ for the conditional probabilities of the hidden variables.

For modeling a TFBS $\underline{x}$ of length w, we use a position weight matrix (PWM, [1, 2]), which assumes that all nucleotides at all positions are statistically independent of each other. Using a Markov Random Field parameterization [3, 4] that is well-suited for numerical optimization, the likelihood of a PWM model can be written as

$$P^{\mathrm{PWM}}\left(\underline{x}\middle|\underline{\lambda}^{(\mathrm{PWM})}\right) := \frac{\exp\left(\sum_{\ell=1}^{w}\lambda_{\ell,x_\ell}^{(\mathrm{PWM})}\right)}{Z\left(\underline{\lambda}^{(\mathrm{PWM})}\right)}, \tag{1a}$$

where

$$Z\left(\underline{\lambda}^{(\mathrm{PWM})}\right) := \sum_{\underline{x}} \exp\left(\sum_{\ell=1}^{w}\lambda_{\ell,x_\ell}^{(\mathrm{PWM})}\right) \tag{1b}$$

is a normalization constant. Since TFBSs can be located on either strand of the DNA, they are often modeled by a mixture model that takes both strands into account

$$P^{\mathcal{M}}\left(\underline{x}\middle|\underline{\lambda}^{(\mathcal{M})}\right) := P^{\mathcal{M}}\left(u=0\middle|\underline{\lambda}_m^{(\mathcal{M})}\right)\cdot P^{\mathrm{PWM}}\left(\underline{x}\middle|\underline{\lambda}^{(\mathrm{PWM})}\right) + P^{\mathcal{M}}\left(u=1\middle|\underline{\lambda}_m^{(\mathcal{M})}\right)\cdot P^{\mathrm{PWM}}\left(\underline{x}^{RC}\middle|\underline{\lambda}^{(\mathrm{PWM})}\right) \tag{1c}$$

with

$$P^{\mathcal{M}}\left(u\middle|\underline{\lambda}_m^{(\mathcal{M})}\right) := \frac{\exp\left(\lambda_u^{(\mathcal{M})}\right)}{\sum_{i=0}^{1}\exp\left(\lambda_i^{(\mathcal{M})}\right)}, \tag{1d}$$

where $\underline{\lambda}_m^{(\mathcal{M})} := \left(\lambda_0^{(\mathcal{M})}, \lambda_1^{(\mathcal{M})}\right)$ and $\underline{\lambda}^{(\mathcal{M})} := \left(\underline{\lambda}_m^{(\mathcal{M})}, \underline{\lambda}^{(\mathrm{PWM})}\right)$.

In analogy to Improbizer [5] or A-GLAM [6], we define a position model that allows to score the start position of a TFBS relative to an anchor position. Although modeling the positional preference exactly is a desirable goal, it must be modeled carefully to avoid overfitting. Both Improbizer and A-GLAM use a Gaussian distribution for modeling the positional preference, assuming that TFBSs are nonuniformly but symmetrically distributed about some mean value. The Gaussian model often works better than a uniform model, but sometimes the TFBSs are not symmetrically distributed about the mean, especially if the mean value is close to the anchor point. Additionally, it might be problematic to use a Gaussian distribution if some BSs are far apart from the mean of the Gaussian. Hence, we choose a mixture distribution of two components for modeling the position $\ell \in [L_0, L_1]$[1]

[1] The current implementation of Dispom requires all sequences to be of the same length

$$P^{\mathcal{S}}\left(\ell\middle|\underline{\lambda}^{(\mathcal{S})}\right) := \sum_{u=1}^{2} P^{\mathcal{S}}\left(u\middle|\underline{\lambda}_m^{(\mathcal{S})}\right)\cdot P^{\mathcal{S}_u}\left(\ell\middle|\underline{\lambda}^{(\mathcal{S}_u)}\right) \tag{2a}$$

where the probability of process u denoted by $P^{\mathcal{S}}\left(u\middle|\underline{\lambda}_m^{(\mathcal{S})}\right)$ is defined in close analogy to Eqn. (1d) as

$$P^{\mathcal{S}}\left(u\middle|\underline{\lambda}_m^{(\mathcal{S})}\right) := \frac{\exp\left(\lambda_u^{(\mathcal{S})}\right)}{\sum_{i=0}^{1}\exp\left(\lambda_i^{(\mathcal{S})}\right)}. \tag{2b}$$

Here $\underline{\lambda}_m^{(\mathcal{S})} := \left(\lambda_0^{(\mathcal{S})}, \lambda_1^{(\mathcal{S})}\right)$ and $\underline{\lambda}^{(\mathcal{S})} := \left(\underline{\lambda}_m^{(\mathcal{S})}, \underline{\lambda}^{(\mathcal{S}_0)}, \underline{\lambda}^{(\mathcal{S}_1)}\right)$. To simplify the further notation, we define $\Delta := L_1 - L_0 > 0$.

The first component $\mathcal{S}_0$ is a uniform distribution

$$P^{\mathcal{S}_0}(\ell) := \frac{1}{\Delta}. \tag{2c}$$

The second component a discrete distribution that is related to the skew normal distribution [7]. The likelihood for this distribution is given by

$$P^{\mathcal{S}_1}\left(\ell\middle|\underline{\lambda}^{(\mathcal{S}_1)}\right) := \frac{s^{\mathcal{S}_1}\left(\ell\middle|\underline{\lambda}^{(\mathcal{S}_1)}\right)}{Z^{\mathcal{S}_1}(\underline{\lambda}^{(\mathcal{S}_1)})} \tag{2d}$$

with the normalization constant

$$Z^{\mathcal{S}_1}(\underline{\lambda}^{(\mathcal{S}_1)}) := \sum_{l=L_0}^{L_1} s^{\mathcal{S}_1}\left(\ell\middle|\underline{\lambda}^{(\mathcal{S}_1)}\right), \tag{2e}$$

and

$$s^{\mathcal{S}_1}\left(\ell\middle|\underline{\lambda}^{(\mathcal{S}_1)}\right) := \exp\left(-0.5\cdot g\left(\ell, \lambda_0^{(\mathcal{S}_1)}, \exp\left(-0.5\lambda_1^{(\mathcal{S}_1)}\right)\right)^2\right)\cdot\phi\left(\lambda_2^{(\mathcal{S}_1)}\cdot g\left(\ell, \lambda_0^{(\mathcal{S}_1)}, \exp\left(-0.5\lambda_1^{(\mathcal{S}_1)}\right)\right)\right) \tag{2f}$$

where g is the auxiliary function

$$g\left(\ell, \lambda_0^{(\mathcal{S}_1)}, \tilde{\sigma}\right) := \frac{\ell - \left[L_0 + \Delta\cdot\left[0.01\lambda_0^{(\mathcal{S}_1)} + \frac{\exp\left(\lambda_0^{(\mathcal{S}_1)}\right)}{1+\exp\left(\lambda_0^{(\mathcal{S}_1)}\right)}\right]\right]}{\tilde{\sigma}}, \tag{2g}$$

and ϕ is the cumulative distribution function of the standard normal distribution. The skew normal distribution and its discrete counterpart constitute a family of distributions with only three parameters that contains the uniform (if $\lambda_1^{(\mathcal{S}_1)} \to -\infty$) and the normal distribution (if $\lambda_2^{(\mathcal{S}_1)} = 0$) as special cases, yet allows sufficient flexibility to adapt to non-symmetric cases.

For modeling the DNA sequences flanking the TFBSs, we use a homogeneous Markov model of order 0, which assumes that the nucleotides are statistically independent and identically distributed at all positions of the sequence, yielding the likelihood

$$P^{\mathcal{F}}\left(\underline{x}\middle|\underline{\lambda}^{(\mathcal{F})}\right) := \prod_{\ell=1}^{L} P^{\mathcal{F}}\left(x_\ell\middle|\underline{\lambda}^{(\mathcal{F})}\right). \tag{3a}$$

Following [8], we define the distribution using the parameter vector $\underline{\lambda}^{(\mathcal{F})}$as

$$P^{\mathcal{F}}\left(b\middle|\underline{\lambda}^{(\mathcal{F})}\right) := \frac{\exp\left(\lambda_b^{(\mathcal{F})}\right)}{\sum_{\tilde{b}\in\Sigma}\exp\left(\lambda_{\tilde{b}}^{(\mathcal{F})}\right)}. \tag{3b}$$

However, the object oriented implementation of Dispom within the framework of Jstacs (http://www.jstacs.de) enables the user to easily exchange the component models $\mathcal{M}$, $\mathcal{S}$, and $\mathcal{F}$.

Appendix B: Prior

When learning the parameters using a Bayesian learning principle as for instance maximum a posteriori, MAP, or maximum supervised posterior, MSP, we need a prior density for the parameters. In this section we describe the prior used in in Dispom. Similar to the modularity of Dispom, we use a modular prior for the parameters.

- For the parameters of PWM model, we use a generalized Dirichlet prior [9]

$$Q^{\mathrm{PWM}}\left(\underline{\lambda}^{(\mathrm{PWM})}\middle|\underline{\alpha}^{(\mathrm{PWM})}\right) \propto Z^{\mathrm{PWM}}\left(\underline{\lambda}^{(\mathrm{PWM})}\right)^{-\alpha_\cdot^{(\mathrm{PWM})}} \cdot \exp\left(\sum_{\ell=1}^{w}\sum_{b\in\Sigma}\alpha_{\ell,b}^{(\mathrm{PWM})}\lambda_{\ell,b}^{(\mathrm{PWM})}\right), \tag{4a}$$

  where for $\ell \in [1,w]:\ \alpha_\cdot^{(\mathrm{PWM})} := \sum_{b\in\Sigma}\alpha_{\ell,b}^{(\mathrm{PWM})}$.

- For the parameters of the strand probabilities of the position distribution $\mathcal{S}$, we use a Dirichlet in softmax notation [8]

$$Q\left(\underline{\lambda}_m^{(\mathcal{M})}\middle|\underline{\alpha}_m^{(\mathcal{M})}\right) \propto \frac{\exp\left(\sum_{u=0}^{1}\alpha_u^{(\mathcal{M})}\lambda_u^{(\mathcal{M})}\right)}{\left(\sum_{u=0}^{1}\exp\left(\alpha_u^{(\mathcal{M})}\lambda_u^{(\mathcal{M})}\right)\right)^{\alpha_\cdot^{(\mathcal{M})}}} \tag{4b}$$

  with $\alpha_\cdot^{(\mathcal{M})} := \alpha_0^{(\mathcal{M})} + \alpha_1^{(\mathcal{M})}$ and $\alpha_\cdot^{(\mathcal{M})} = \alpha_\cdot^{(\mathrm{PWM})}$.

- For the parameters of the mixture probabilities, we use a Dirichlet in softmax notation [8]

$$Q\left(\underline{\lambda}_m^{(\mathcal{S})}\middle|\underline{\alpha}_m^{(\mathcal{S})}\right) \propto \frac{\exp\left(\sum_{u=0}^{1}\alpha_u^{(\mathcal{S})}\lambda_u^{(\mathcal{S})}\right)}{\left(\sum_{u=0}^{1}\exp\left(\alpha_u^{(\mathcal{S})}\lambda_u^{(\mathcal{S})}\right)\right)^{\alpha_\cdot^{(\mathcal{S})}}} \tag{4c}$$

  with $\alpha_\cdot^{(\mathcal{S})} := \alpha_0^{(\mathcal{S})} + \alpha_1^{(\mathcal{S})}$ and $\alpha_1^{(\mathcal{S})} = \alpha_\cdot^{(\mathrm{PWM})}$.

- For the parameters of the discrete skew normal distribution, we separate priors for each parameter.
  - For the first parameter $\lambda_0^{(\mathcal{S}_1)}$, which is related to the mean of the distribution, we choose a transformed Gaussian distribution.

$$Q_0^{\mathcal{S}_1}\left(\lambda_0^{(\mathcal{S}_1)}\middle|\underline{\alpha}_0^{(\mathcal{S}_1)}\right) \propto \exp\left(-0.5 \cdot g\left(\alpha_{0,0}^{(\mathcal{S}_1)}, \lambda_0^{(\mathcal{S}_1)}, \alpha_{0,1}^{(\mathcal{S}_1)}\right)^2\right) \cdot \left[0.01 + \frac{\exp\left(\lambda_0^{(\mathcal{S}_1)}\right)}{\left[1+\exp\left(\lambda_0^{(\mathcal{S}_1)}\right)\right]^2}\right] \tag{4d}$$

  - For the second parameter $\lambda_1^{(\mathcal{S}_1)}$, which is related to the standard deviation of the distribution, we choose a transformed Gamma distribution.

$$Q_1^{\mathcal{S}_1}\left(\lambda_1^{(\mathcal{S}_1)}\middle|\underline{\alpha}_1^{(\mathcal{S}_1)}\right) \propto \exp\left(\alpha_{1,0}^{(\mathcal{S}_1)}\lambda_1^{(\mathcal{S}_1)} - \alpha_{1,1}^{(\mathcal{S}_1)}\exp\left(\alpha_1^{(\mathcal{S}_1)}\right)\right) \tag{4e}$$

  - For the third parameter $\lambda_2^{(\mathcal{S}_1)}$, which is related to the skew of the distribution, we choose a Gaussian distribution.

$$Q_2^{\mathcal{S}_1}\left(\lambda_2^{(\mathcal{S}_1)}\middle|\underline{\alpha}_2^{(\mathcal{S}_1)}\right) \propto \exp\left(-0.5 \cdot \left(\frac{\lambda_2^{(\mathcal{S}_1)} - \alpha_{2,0}^{(\mathcal{S}_1)}}{\alpha_{2,1}^{(\mathcal{S}_1)}}\right)^2\right) \tag{4f}$$

- For the parameters of the homogeneous Markov model, we use a Dirichlet in softmax notation [8]

$$Q\left(\underline{\lambda}_m^{(\mathcal{F})}\middle|\underline{\alpha}_m^{(\mathcal{F})}\right) \propto \frac{\exp\left(\sum_{b\in\Sigma}\alpha_b^{(\mathcal{F})}\lambda_b^{(\mathcal{F})}\right)}{\left(\sum_{b\in\Sigma}\exp\left(\alpha_b^{(\mathcal{F})}\lambda_b^{(\mathcal{F})}\right)\right)^{\alpha_\cdot^{(\mathcal{F})}}} \tag{4g}$$

  with $\alpha_\cdot^{(\mathcal{F})} := \sum_{b\in\Sigma}\alpha_b^{(\mathcal{F})}$.

In the cases studies on the benchmark data sets as well as for the auxin data set, we used

- $\alpha_{\ell,b}^{(\mathrm{PWM})} = 1$ for $\ell \in [1, w], b \in \Sigma$,
- $\alpha_b^{(\mathcal{M})} = 2$ for $b \in \{0, 1\}$,
- $\alpha_0 = 1$,
- $\alpha_0^{\mathcal{S}} = 3$ and $\alpha_1^{\mathcal{S}} = 1$,
- $\alpha_{0,0}^{(\mathcal{S}_1)} = 250$ and $\alpha_{0,1}^{(\mathcal{S}_1)} = 500$, $\alpha_{1,0}^{(\mathcal{S}_1)} = 0.5$ and $\alpha_{1,1}^{(\mathcal{S}_1)} = 0.5 \cdot 150^2$, $\alpha_{2,0}^{(\mathcal{S}_1)} = 0$ and $\alpha_{2,1}^{(\mathcal{S}_1)} = 1$, and
- $\alpha_b^{(\mathcal{F})} = L \cdot \frac{\alpha_0 + \alpha_\cdot^{(\mathrm{PWM})}}{4} - \sum_{\ell=1}^{w}\alpha_{\ell,b}^{(\mathrm{PWM})}$ for $b \in \Sigma$ which is initial 610.

Appendix C: Heuristic

In the methods section, we briefly describe the heuristics used in Dispom. Here, we describe the procedure for proposing a promising modification of the motif model in more detail. In addition, we provide Figure I that visualizes the heuristic.

First, we compute the number of foreground sequences B predicted to contain at least one BS. Second, we test each shift s of the motif of at most half of the motif length w as follows: We shift the motif model to the right by copying the current parameter values of the PWM s positions to the left, i.e. $\forall \ell = 1, \ldots, w-s, \forall a \in \Sigma : \lambda^{(\mathrm{PWM})}_{\ell,a} := \lambda^{(\mathrm{PWM})}_{\ell+s,a}$. Since we have no parameter values for positions $w+1$ and above that could be copied to positions $w-s+1$ and above, we initialize those positions by a uniform distribution, i.e. $\forall \ell = w-s+1, \ldots, w : \forall a \in \Sigma : \lambda^{(\mathrm{PWM})}_{\ell,a} := 0$. We conduct shifts to the left in complete analogy. After shifting the motif model, we optimize the parameters by 10 steps of numerical optimization according to Equation 4, and compute the number of foreground sequences B_s

(a) Flow diagram for Dispom.

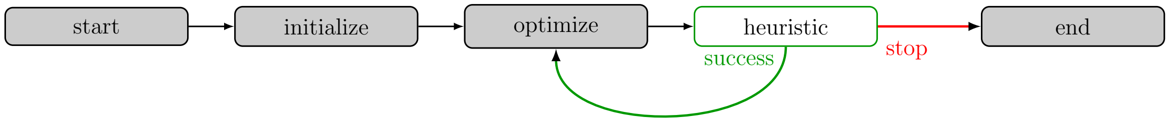

(b) Flow diagram for the heuristic used in Dispom.

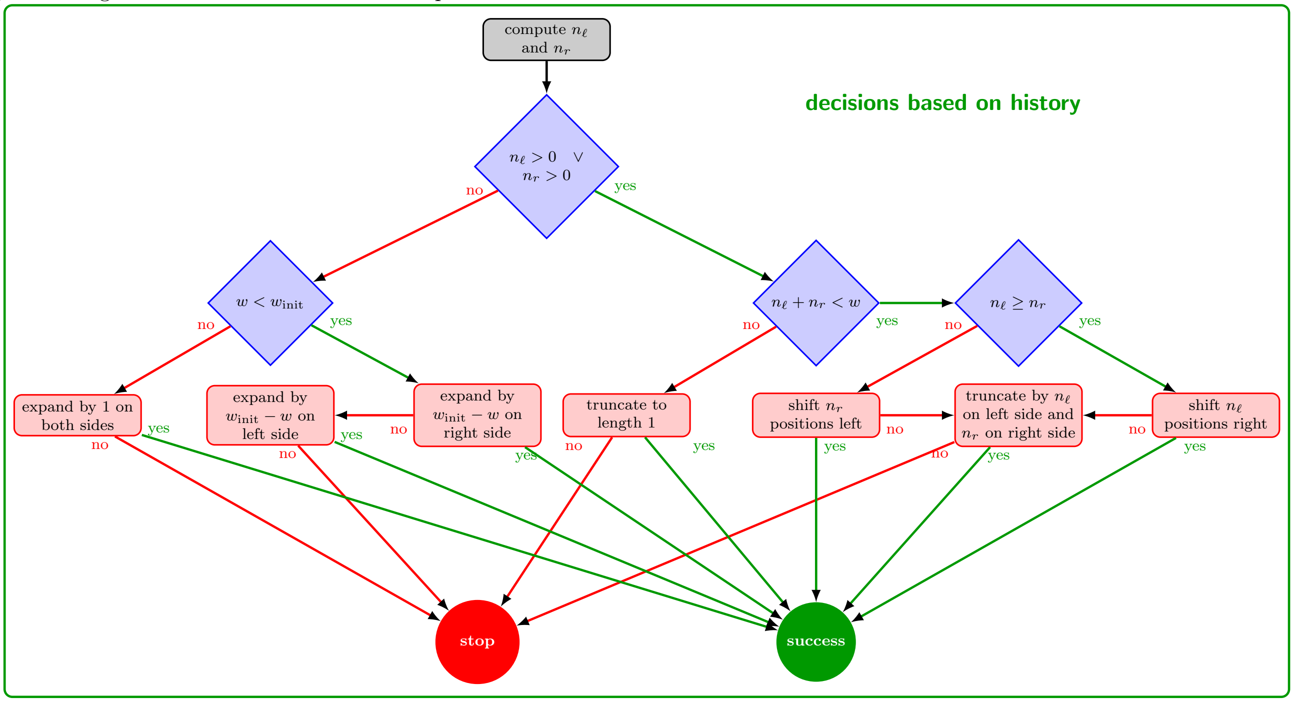

Figure I: Flow diagram for Dispom. Figure a) shows the flow diagram for Dispom, whereas Figure b) shows the flow diagram of the heuristic that is used to shift, truncate, or expand the motif.

predicted to contain at least one BS. Finally, we compare the B and B_s, and determine for both shift directions the number of insignificant positions by finding the shift $s^* = \max_s \{s|B_s \geq 0.8 \cdot B\}$. From these insignificant positions, the heuristic proposes a promising modification of the motif model.

The promising modifications are determined by the following rules: Let n_ℓ be the number of insignificant positions on the left side of the motif, and let n_r be the number of insignificant positions on the right side of the motif, i.e. n_ℓ is the s^* determined by shifting the motif model to the right and n_r is the s^* determined by shifting the motif model to the left. If we find no insignificant position on either side of the motif, i.e. $n_\ell = n_r = 0$ we expand the motif to the initial length by appending additional positions to the right side, or if this configuration is already stored in the history, to the left side of the motif. If the initial length is already reached or even exceeded, we expand the motif by one position on both sides, if allowed by the history. Otherwise, i.e. $n_\ell > 0$ or $n_r > 0$, we first try to shift the motif, such that the larger number of insignificant positions is shifted out of the model, i.e. if $n_\ell > n_r$, we shift the model by n_ℓ positions to the right, and vice versa. If the shift operation did not succeed, we truncate the motif by removing n_ℓ positions from the left side and n_r positions from the right side of the motif. We restrict the minimal length of the motif model to 1 to prevent the complete elimination of the motif. Positions that are added to the motif model are initialized with a uniform distribution of nucleotides before we start the numerical optimization. The cycle of heuristic steps and consequent optimization is stopped if none of the promising modifications is still allowed by the history. Figure I shows the complete flow diagram of Dispom as well as a detailed workflow for the heuristic described above.

Appendix D: Common sequence patterns

To assess the significance of predictions depending on the amount of data, we estimate the probability to find at least one common subsequence with at most one mismatch in N random sequences. We download Arabidopsis promoter regions from TAIR [10] and extract the upstream 2000 bp for each promoter following [6]. For varying N, we randomly sample N promoter sequences and determine the length of the longest common subsequence of the N sequences with at most one mismatch. In Figure II, we show the result of repeating this procedure 1000 times where we plot the length of the longest subsequence against the p-value. For N equal to 5, 10, and 100, and a subsequence length of upto 10, 9, and 8 bp, respectively, we find a p-value of 1.[2] Using less conservative approaches as for instance the ZOOPS model, we are not restricted to one mismatch per BS, and we do not require that each sequence contains a BS, making the problem even worse. Hence, finding binding motifs of length 9 bp in data sets with few long sequences is usually insignificant, and we recommend to use benchmark data sets with more or shorter sequences.

[2] We perform the same simulation for human promoters obtaining similar results.

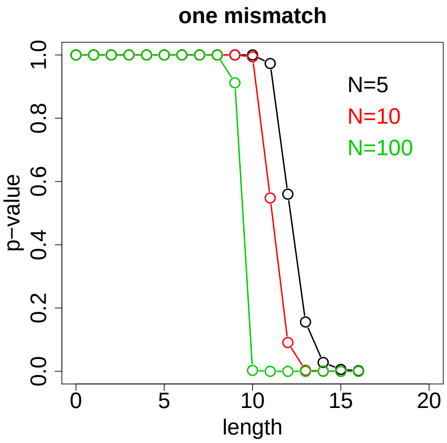

Figure II: P-value distribution for the length of common subsequences in promoter regions of *Arabidopsis thaliana*. Illustration of the p-value distribution for the length of common subsequences in promoter regions of *Arabidopsis thaliana* for N equals 5, 10, and 100 sequences, respectively, and one allowed mismatch.

Appendix E: Runtime comparison

We compare the runtime of Dispom to that of other tools, namely A-GLAM, DEME, DME, Gibbs Sampler, Improbizer, MEME, and Weeder, on a set of example data sets. We generate these data sets by sub-sampling 50,

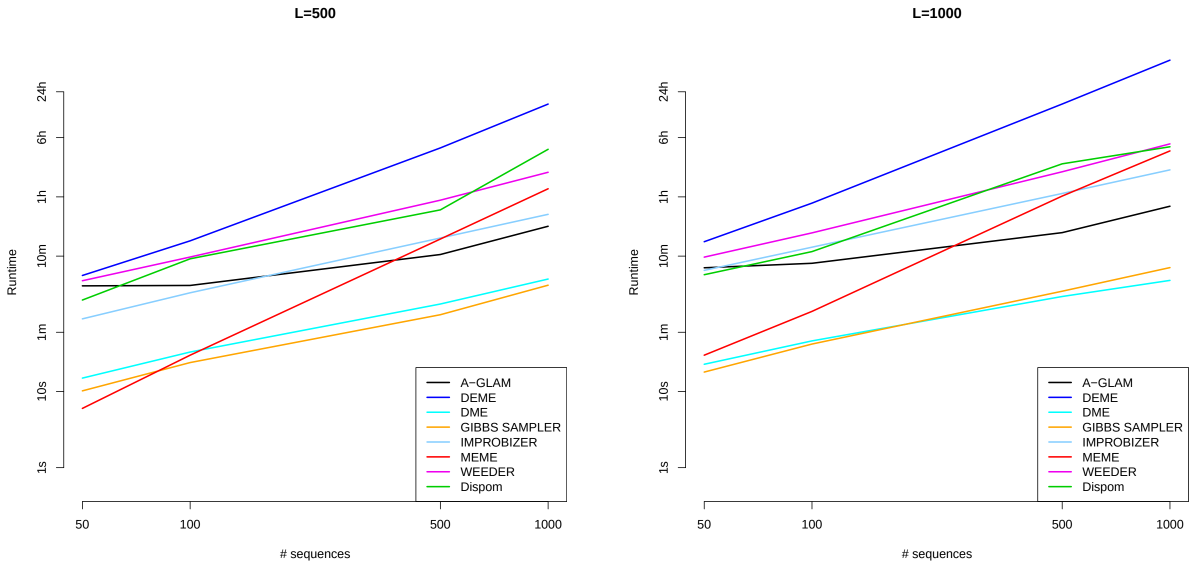

(a) (b)

Figure III: Runtime comparison of Dispom and other tools on data sets comprising different numbers of sequences of length $L = 500$ (a) and length $L = 1000$ (b).

100, 500, and 1000 promoters of the data set "Elegans-GATA-Pauli-1427" of the metazoan compendium. For those tools that make use of a control data set, we sample another data set of the same size. To also consider the length of the input sequences in the comparison, we cut the sequences to i) 500 bp upstream of the TSS and ii) 1000 bp upstream of the TSS.

For each of the tools, we measure the runtime of a single start of each tool using a fixed motif length of 10 bp. We present the results of the runtime comparison in Figure III. In general, we observe great differences in the runtimes of the tools considered. In all cases, DME and Gibbs Sampler are among the tools with the shortest runtime, whereas Weeder, DEME, and Dispom require the most computation time.

The runtime of Dispom highly depends on the number of heuristic steps performed to compensate for phase shifts, since the numerical optimization is restarted after each heuristic step. Hence, the runtime of Dispom may differ from the results on the example data sets, depending on the specific data set and the heuristic steps allowed.

Appendix F: Calls of de-novo motif discovery tools

In the following, we specify the calls of the de-novo discovery tools used in the benchmark studies on the BS level. The placeholder `<target>` represents the target data set, `<control>` represents the control data set, and `<length>` represents the length of the correct motif. For tools that provide several motifs, we only use the first motif.

A-GLAM [6]

unknown length:

```
./aglam -4 500 <target>
```

known length:

```
./aglam -a <length> -b <length> -4 500 <target>
```

Meaning of additional arguments:

- `-4` anchor position of the position distribution
- `-a, -b` minimum and maximum length of the motif

DEME [11]

unknown length:

```
./deme -p <target> -n <control> -w 15
```

known length:

```
./deme -p <target> -n <control> -w <length>
```

Meaning of additional arguments:

- `-w` length of the motif

DME [12]

unknown length:

```
./dme2 -v -n 200 -w 15 -o <outfile> -b <control> <target>
```

known length:

```
./dme2 -v -n 200 -w <length> -o <outfile> -b <control> <target>
```

Meaning of additional arguments:

- `-o` followed by path to the output-file `<outfile>`
- `-v` verbose output
- `-n` number of motifs to produce

Gibbs sampler [13]

unknown length:

```
./Gibbs <target> 15 -n
```

known length:

```
./Gibbs <target> <length> -n
```

Meaning of additional arguments:

- `-n` use nucleic acid alphabet

Improbizer [5]

unknown length:

```
./ameme good=<target> bad=<control> numMotifs=1 rcToo=on \
        motifOutput=<target>-motif.txt
./ameme motifMatcher=on seqFile=<target> rcToo=on \
        motifs=<target>-motif.txt hits=<target>-hits.txt
```

known length:

```
./ameme good=<target> bad=<control> numMotifs=1 rcToo=on constrainer=1000 \
        tileSize=<length> motifOutput=<target>-motif.txt
./ameme motifMatcher=on seqFile=<target> rcToo=on \
        motifs=<target>-motif.txt hits=<target>-hits.txt
```

Meaning of additional arguments:

- `numMotifs` number of motifs
- `tileSize` length of motif
- `constrainer=1000` fix motif length
- `motifMatcher=on` predict motif occurrences
- `rcToo=on` search on both strands

MEME [14]
unknown length:

```
./meme -dna -mod zoops -minw 6 -maxw 20 -nmotifs 1 -revcomp -text <target>
```

known length:

```
./meme -dna -mod zoops -w <length> -nmotifs 1 -revcomp -text <target>
```

Meaning of additional arguments:

- `-dna` use nucleic acid alphabet
- `-mod zoops` use ZOOPS model
- `-minw,-maxw` minimum and maximum motif length
- `-w` motif length
- `-nmotifs` number of motifs
- `-revcomp` search on both strands
- `-text` text output instead of HTML

Weeder [15]
unknown length:

```
./weederlauncher.out <target> <organism> large S
```

known length:

```
./weederTFBS.out -f <target> -R 50 -O <organism> -W <length> -e 3 -S -T 10
./adviser.out <target> S
```

Meaning of additional arguments:

- `-O <organism>` organism, AT for *A. thaliana*, DM for *D. melanogaster*, HS for *H. sapiens*
- `large` search for motifs of maximum length 12 with at most 4 mismatches
- `S,-S` search on both strands
- `-R 50` percentage of sequence that must contain the motif
- `-W` length of the motif
- `-e` number of allowed mismatches
- `-T` number of reported motifs

Appendix G: Evaluating the ZOOPS model assumption

Many currently available de-novo motif discovery tools are based on the ZOOPS model, which assumes that there is zero or one BS per sequence. As this assumption is often unrealistic, we evaluate for two data sets to which degree this assumption hampers Dispom in finding the motif and predicting individual BSs.

In complete analogy to data set MA0048, we construct four data sets that are not based on the assumptions of the ZOOPS model by implanting either zero or two BSs (data sets 1 and 2) or zero to five BSs (data sets 3 and 4) per sequence using either a uniform (data sets 1 and 3) or a Gaussian (data sets 2 and 4) positional distribution. We

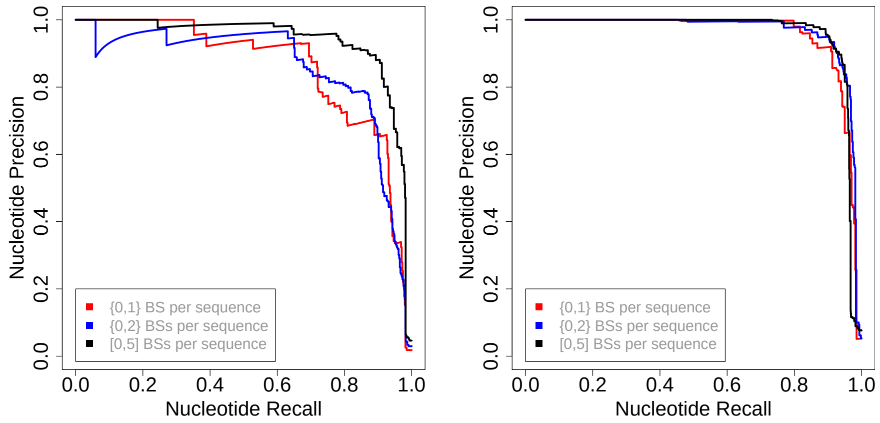

(a) Uniform distributed BSs (b) Gaussian distributed BSs

# BSs	Uniform	Gaussian
$\{0,1\}$	0.861	0.955
$\{0,2\}$	0.856	0.964
$[0,5]$	0.938	0.958

(c) AUC-PR

Figure IV: The Figure shows the nucleotide precision recall curves and the AUC-PR for Dispom based on data sets for the TF MA0048. The left (middle) panel shows the the performance of Dispom for uniformly (Gaussian) distributed BSs. The red curve indicates the performance of Dispom for at most one implanted BS per sequence, while the blue and the black curves indicate the performance for 0 or 2 BSs and 0 to 5 BSs per sequence, respectively. The right panel shows the AUC-PR for the curves of the left an the right panel. We find that Dispom is not seriously affected by the violation of the assumptions of the ZOOPS model.

run Dispom on these data sets, let it predict BSs for each data set, and show the resulting four nucleotide precision recall curves together in Figure IV. We find that Dispom performs well in all four cases, indicating that Dispom is not badly hampered by the presence of multiple binding sites.

References

[1] Stormo G, Schneider T, Gold L, Ehrenfeucht A (1982) Use of the 'perceptron' algorithm to distinguish translational initiation sites. Nucleic Acids Res 10: 2997-3010.

[2] Staden R (1984) Computer methods to locate signals in nucleic acid sequences. Nucleic Acids Res 12: 505-519.

[3] Berger A, Della Pietra S, Della Pietra V (1996) A maximum entropy approach to natural language processing. Computational Linguistics 22: 39–71.

[4] Klein D, Manning C (2003). Maxent models, conditional estimation, and optimization. HLT-NAACL 2003 Tutorial. URL `http://www.cs.berkeley.edu/ klein/papers/maxent-tutorial-slides.pdf`.

[5] Ao W, Gaudet J, Kent WJ, Muttumu S, Mango SE (2004) Environmentally Induced Foregut Remodeling by PHA-4/FoxA and DAF-12/NHR. Science 305: 1743-1746.

[6] Kim NK, Tharakaraman K, Marino-Ramirez L, Spouge JL (2008) Finding sequence motifs with Bayesian models incorporating positional information: an application to transcription factor binding sites. BMC Bioinformatics 9: 262+.

[7] Azzalini A (1985) A class of distributions which includes the normal ones. Scandinavian journal of statistics 12: 171-178.

[8] MacKay DJC (1998) Choice of basis for Laplace approximation. Mach Learning 33: 77-86.

[9] Keilwagen J, Grau J, Posch S, Grosse I (2009) Apples and oranges: avoiding different priors in Bayesian DNA sequence analysis. BMC Bioinformatics Submitted.

[10] Swarbreck D, Wilks C, Lamesch P, Berardini TZ, Garcia-Hernandez M, et al. (2008) The Arabidopsis Information Resource (TAIR): gene structure and function annotation. Nucleic Acids Res 36: D1009–D1014.

[11] Redhead E, Bailey TL (2007) Discriminative motif discovery in DNA and protein sequences using the DEME algorithm. BMC Bioinformatics 8: 385.

[12] Smith AD, Sumazin P, Zhang MQ (2005) Identifying tissue-selective transcription factor binding sites in vertebrate promoters. Proc Natl Acad Sci U S A 102: 1560-1565.

[13] Lawrence CE, Altschul SF, Boguski MS, Liu JS, Neuwald AF, et al. (1993) Detecting subtle sequence signals: A Gibbs sampling strategy for multiple alignment. Science 262: 208-214.

[14] Bailey TL, Elkan C (1994) Fitting a mixture model by expectation maximization to discover motifs in biopolymers. In: Proceedings of the Second International Conference on Intelligent Systems for Molecular Biology; 14-17 August 1994; Stanford, California, United States. AAAI Press, pp. 28-36. URL `http://www.sdsc.edu/ tbailey/papers/ismb94.ps`.

[15] Pavesi G, Mauri G, Pesole G (2001) An algorithm for finding signals of unknown length in DNA sequences. Bioinformatics 17: S207-214.
